# Sorcin is an early marker of neurodegeneration, Ca^2+^ dysregulation and endoplasmic reticulum stress associated to neurodegenerative diseases

**DOI:** 10.1038/s41419-020-03063-y

**Published:** 2020-10-15

**Authors:** Ilaria Genovese, Flavia Giamogante, Lucia Barazzuol, Theo Battista, Annarita Fiorillo, Mattia Vicario, Giuseppina D’Alessandro, Raffaela Cipriani, Cristina Limatola, Daniela Rossi, Vincenzo Sorrentino, Elena Poser, Luciana Mosca, Ferdinando Squitieri, Marzia Perluigi, Andrea Arena, Filip van Petegem, Claudia Tito, Francesco Fazi, Carlotta Giorgi, Tito Calì, Andrea Ilari, Gianni Colotti

**Affiliations:** 1 Department of Medical Sciences, Laboratory for Technology of Advanced Therapies (LTTA) University of Ferrara, Ferrara, Italy; 2 Department of Biomedical Sciences, University of Padova, Padova, Italy; 3 Padova Neuroscience Center (PNC), University of Padova, Padova, Italy; 4 Department of Biochemical Sciences “A. Rossi Fanelli”, University Sapienza of Rome, Rome, Italy; 5 Department of Physiology and Pharmacology, University of Rome “Sapienza”, Rome, Italy; 6 IRCCS Neuromed, Pozzilli, Isernia, Italy; 7 Department of Physiology and Pharmacology, Sapienza University, Laboratory Affiliated to Istituto Pasteur Italia – Rome, Rome, Italy; 8 Department of Molecular and Developmental Medicine, University of Siena, Siena, Italy; 9 Department of Biochemistry, University of Oxford, Oxford, UK; 10 Huntington’s and Rare Diseases Unit, IRCCS Ospedale Casa Sollievo della Sofferenza, Rome, Italy; 11 Department of Biochemistry and Molecular Biology, Faculty of Medicine, University of British Columbia, Vancouver, Canada; 12 Department of Anatomical, Histological, Forensic & Orthopedic Sciences, Section of Histology & Medical Embryology, Sapienza University of Rome, Laboratory affiliated to Istituto Pasteur Italia-Fondazione Cenci Bolognetti, Rome, Italy; 13 Institute of Molecular Biology and Pathology, Italian National Research Council, IBPM-CNR, Rome, Italy

**Keywords:** Calcium signalling, Stress signalling, Mechanisms of disease, Huntington's disease

## Abstract

Dysregulation of calcium signaling is emerging as a key feature in the pathogenesis of neurodegenerative diseases such as Alzheimer’s disease (AD), Parkinson’s disease (PD), and Huntington’s disease (HD), and targeting this process may be therapeutically beneficial. Under this perspective, it is important to study proteins that regulate calcium homeostasis in the cell. Sorcin is one of the most expressed calcium-binding proteins in the human brain; its overexpression increases endoplasmic reticulum (ER) calcium concentration and decreases ER stress in the heart and in other cellular types. Sorcin has been hypothesized to be involved in neurodegenerative diseases, since it may counteract the increased cytosolic calcium levels associated with neurodegeneration. In the present work, we show that Sorcin expression levels are strongly increased in cellular, animal, and human models of AD, PD, and HD, vs. normal cells. Sorcin partially colocalizes with RyRs in neurons and microglia cells; functional experiments with microsomes containing high amounts of RyR2 and RyR3, respectively, show that Sorcin is able to regulate these ER calcium channels. The molecular basis of the interaction of Sorcin with RyR2 and RyR3 is demonstrated by SPR. Sorcin also interacts with other ER proteins as SERCA2 and Sigma-1 receptor in a calcium-dependent fashion. We also show that Sorcin regulates ER calcium transients: Sorcin increases the velocity of ER calcium uptake (increasing SERCA activity). The data presented here demonstrate that Sorcin may represent both a novel early marker of neurodegenerative diseases and a response to cellular stress dependent on neurodegeneration.

## Introduction

Calcium is one of the most important signaling factors in the human body, regulating important cellular functions, such as differentiation, proliferation, growth, survival, apoptosis, gene transcription, and membrane excitability, and playing a fundamental role especially in excitable cells as neurons and cardiomyocytes.

Many neurodegenerative diseases are characterized by formation of aggregates of misfolded or mutated proteins/peptides, such as Aβ peptides in Alzheimer’s disease (AD), α-synuclein (αSyn) in Parkinson’s disease (PD), and mutated huntingtin (mHtt) in Huntington’s disease (HD); however, calcium dysregulation is associated with all these diseases and in many cases precedes the detectable pathologies, such that a “calcium hypothesis of brain aging and AD” was proposed^1,2^. Dysregulated calcium homeostasis is an early pathogenic event that promotes amyloidogenesis, protein aggregation, neuronal energy deficits and oxidative stress, cytoskeletal alterations, mitochondrial dysfunction, synaptic transmission and plasticity dysfunction, and other age-related features such as impaired lysosomal function, defects in repair mechanisms and altered response to metabolic challenges or cellular stress^3^.

**Fig. 1:**
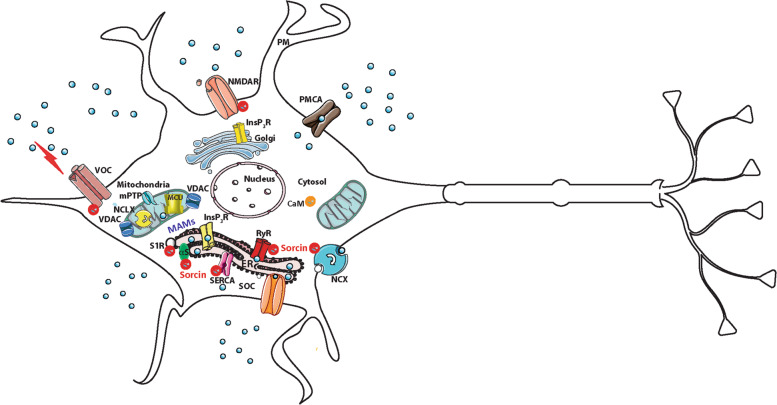
Sorcin and calcium homeostasis. Sorcin interacts with many calcium channels, pumps and exchangers involved in calcium homeostasis, and with other proteins involved in neurodegeneration, apoptosis and glucose tolerance. Many proteins are involved in Ca^2+^ homeostasis, such as the inositol 1,4,5-trisphosphate receptor (InsP_3_R), the ryanodine receptors (RyRs) at the sarco/endoplasmic reticulum (SR/ER) membranes, the voltage (VOC), and the store-operated (SOC) Ca^2+^ channels of the plasma membrane. Ca^2+^ extrusion depends on the activity of the plasma membrane Ca^2+^ ATPase (PMCA) and the plasma membrane Na^+^/Ca^2+^ exchanger (NCX). Ca^2+^ reuptake in the intracellular stores is operated by the ER/SR Ca^2+^ ATPase (SERCA). The mitochondrial Ca^2+^ handling systems involves MCU, mitochondrial Ca^2+^ uniporter; NCLX, mitochondrial Na^+^/Ca^2+^ exchanger; VDAC, voltage-dependent anion channels; MPTP, mitochondrial permeability transition pore. CaM, calmodulin. Sorcin regulates Ryanodine receptors (RyRs) and Sarco(endo)plasmic reticulum Ca^2+^-ATPase (SERCA): Sorcin decreases ER calcium release by inhibiting RyR and increases calcium entry by activating SERCA, thus increasing Ca^2+^ accumulation in the ER, preventing ER stress, and possibly the unfolded protein response. Sorcin binds and regulate VOC channels, interacts with the N-methyl-D-aspartate receptor (NMDAR) and with RyR in caudate-putamen nucleus, and regulates Na^+^-Ca^2+^ exchanger (NCX). Sorcin also interacts in a calcium-dependent fashion with alpha-synuclein (αS) and presenilin 2 (PS2), proteins involved in PD and AD pathogenesis, respectively, and with the Sigma-1 receptor (S1R, see results), a chaperone protein at the ER which is able to regulate calcium signaling, bioenergetics and ER stress.

Sorcin (soluble resistance-related calcium-binding protein) is a key protein in the ER calcium-dependent cascades and is one of the most expressed calcium-binding proteins in the brain (source: PaxDb), and in many brain cancers, such as anaplastic astrocytoma, oligodendroglioma, and glioblastoma^4–6^. Sorcin is also a key regulator of calcium-induced calcium release in the heart^7–10^. Upon calcium binding, Sorcin undergoes large conformational changes, involving exposure of hydrophobic surfaces, that allow it to interact with calcium channels and exchangers like Ryanodine receptors (RyRs) and Sarco(endo)plasmic reticulum Ca^2+^-ATPase (SERCA): Sorcin decreases ER calcium release by inhibiting RyR and increases calcium entry by activating SERCA, thus increasing Ca^2+^ accumulation in the ER (and mitochondria), preventing ER stress, and possibly the unfolded protein response^7,8,10–22^ (Fig. 1). Sorcin activates ATF6 transcriptional activity while repressing ER stress markers as CHOP and Grp78/BiP; conversely, Sorcin silencing activates apoptotic proteases Grp78/BiP, Bcl-2, Bax, c-jun, c-fos and release of cytochrome c, results in major mitosis and cytokinesis defects, blocks cell cycle progression in mitosis, increases the formation of rounded, polynuclear cells, induces apoptosis, and increases mitochondrial Ca^2+^ concentration in cardiomyocytes^15,23,24^.

Sorcin is able to bind and regulate Cav1 channels (voltage-operated calcium channel, VOC), interacts with the N-methyl-D-aspartate receptor (NMDAR) and with RyR in caudate-putamen nucleus, and regulates Na^+^–Ca^2+^ exchanger (NCX); in addition, it interacts in a calcium-dependent fashion with αSyn and presenilin 2 (PS2), proteins involved in calcium homeostasis and in PD and AD pathogenesis, respectively. Moreover, it interacts with annexins A7/A11, which affect functions of primary astrocytes^25–32^ (Fig. 1).

Proteomic analysis of brain cortical samples shows that Sorcin is overexpressed in brains from AD patients vs. control^33,34^. Further, Sorcin is more expressed in the frontal cortex of asymptomatic AD patients with respect to symptomatic AD patients^35^, and is more expressed in amyloid plaques in sporadic vs. rapidly progressive AD patients, and in AD vs. cerebral amyloid angiopathy patients^36,37^, thereby possibly protecting from acceleration progression that takes place in the aggressive forms of the disease. Sorcin is also overexpressed in frontal cortex tissues from frontotemporal dementia, with respect to control patients, especially in glial cell types^38^.

Sorcin is overexpressed in *substantia nigra* of PD patients vs. controls^39^, and in *substantia nigra pars compacta* pathologically verified PD patients vs. controls^40^, is up-regulated in MPP^+^-treated cells^41^, and in induced pluripotent stem cells (iPSCs) derived from PD patients vs. control cells^42^. Further, Sorcin is among the genes overexpressed in 7 human and mouse models of Huntington’s disease, under the control of the ERSE-I (ER stress response element) promoter upstream Sorcin gene, together with other proteins involved in ER stress and unfolded protein response^43^.

Notwithstanding this body of evidence demonstrating the importance of Sorcin in neurodegenerative diseases, Sorcin was never investigated as a neurodegenerative disease marker and/or player.

The present work aims at understanding whether Sorcin expression and function is involved in the rescue of Ca^2+^ dysregulation and ER stress in neurodegenerative diseases, and whether Sorcin expression results in a pro-survival strategy that helps rescue neurodegeneration-dependent cell damages.

We studied expression and localization of Sorcin in neurons, to assess whether Sorcin is localized at membranes and at the ER, where it regulates calcium fluxes. We performed functional experiments with RyR2- and RyR3-containing microsomes, investigating the ability of Sorcin to regulate both types of ER calcium channels present in different body districts, and SPR experiments of Sorcin with RyR peptides, to understand whether Sorcin interacts with and regulates RyR3, in addition to RyR2, and the molecular basis of the inhibition of RyR2 and RyR3. We then studied Sorcin interaction with ER function/dysfunction players, such as SERCA, and the Sigma-1 receptor, besides its role in the establishment of calcium signaling functional contacts between ER and mitochondria^44^.

We measured Sorcin expression in different neurodegenerative disease models, in order to assess whether Sorcin is overexpressed in these models and is able to respond to ER stress in the brain; in particular, we studied Sorcin expression in different phases of neurodegeneration, to understand whether Sorcin is particularly expressed in the early phases of neurodegenerative diseases and whether it may represent an early marker of neurodegeneration.

We also studied how Sorcin expression affects Ca^2+^ fluxes and concentrations in the cytosol and in ER, in order to demonstrate the role of Sorcin in the early neurodegenerative events.

## Materials and methods

### Cortical neuronal cultures

Cortical neuronal cultures were prepared from the brain of newborn wt mice. Cerebral cortices were chopped and digested in 20 U/ml papain for 40 min at 37 °C. Cells (2.5 × 10^5^ cells per well) were seeded on dishes coated with poly-l-lysine (100 μg/ml) in basal medium Eagle supplemented with 1 mm sodium pyruvate, 30 mm glucose, 0.1% Mito serum extender, 10% FBS, 100 U/ml penicillin, 0.1 mg/ml streptomycin, and 10 mm HEPES–NaOH, pH 7.4. After 4 h, the medium was changed with Neurobasal medium supplemented with 1 mM glutamine, 0.1% Mito serum extender, 2.5% B27, 100 U/ml penicillin, and 0.1 mg/ml streptomycin. After 2 d, AraC (5 μm) was added to avoid the growth of glial cells (astrocytes and microglia). The percentage of neuronal cells obtained with this method is between 80 and 90%, as determined with β-tubulin III staining^45^.

### Immunoprecipitation and western blot analysis on cortical neuronal culture

Cortical Neuronal culture were lysed with RIPAC buffer (0.2 M Tris, 1.5 M NaCl, 5% NP-40, 1 mM CaCl_2_ and protein inhibitors). Protein lysates of cortical neurons were quantified with BCA method and proteins (900 μg) were pre-cleared with Protein G-Sepharose (Sigma Aldrich, 60 min, 4 °C) and further incubated with pre-coupled anti-Sorcin antibody (2 μg^46^) with Protein G-Sepharose beads (30 μl). After 3 h incubation at 4 °C, beads were washed three times with ice-cold buffer and centrifuged. Immunoprecipitated proteins were separated on 5% SDS-polyacrylamide gel and visualized by western blot staining with anti RyR (1:2000, Santa Cruz).

Similar pre-cleared protein lysates were incubated with anti RyR (1:2000, Santa Cruz) pre-coupled with Protein G-Sepharose beads (30 μl). After 3 h incubation at 4 °C, beads were washed three times with ice-cold buffer and centrifuged. Immunoprecipitated proteins were separated on 12% SDS-polyacrylamide gel and visualized by Western blot staining with anti-Sorcin antibody (2 μg^46^). Protein detection was performed through the chemiluminescence assay Immun-Star Western C Kit (Bio-Rad). Densitometric analysis was carried out with Quantity One software (Bio-Rad).

### Immunofluorescence

Coronal brain sections (20 μm) were washed in PBS, blocked (3% normal goat serum in 0.3% Triton X-100) for 1 h at RT and incubated overnight at 4 °C with specific antibodies, RyR (1:500, Santa Cruz), Sorcin (1:200, Santa Cruz), Tuj-1 (1:500, Covance), Isolectin 4 (IB4, Sigma, 1:500). Brain slices were stained with the respective fluorophore-conjugated secondary antibodies (Alexa Fluor, Thermofisher) for 1 h at room temperature and Hoechst for nuclei visualization and analyzed using a fluorescence microscope (Nikon Eclipse). For immunofluorescence analysis on cortical neurons, cells (1.5 × 10^5^ cells per well) were fixed with PFA4% and permeabilized. The same specific antibodies described above were used except for β- tubulin III (1:500, Sigma).

### Protein expression and purification

Human Sorcin was expressed and purified according to Ilari et al.^13^. The cytosolic domain of Sigma-1 receptor (residues 1–138) was cloned in frame with GST in a pGEX4T1 vector, expressed in BL21(DE3) *E.coli* cells (induction was obtained adding 1 mM Isopropyl-β-D-thiogalactoside) and purified using GSTrap HP Amersham Biosciences columns according to the GST Gene Fusion System Handbook, Amersham Biosciences, Code No. 18-1157-58.

### Determination of [3H]ryanodine binding to RyR isoforms as a function of Sorcin concentration

Microsomes were prepared according to the method described previously^47^. Microsomal preparations (60 μg) from control HEK293, RyR2, and RyR3-expressing cells were incubated for 1.5 hours at 36 °C with 9 nM [3H]ryanodine in 200 μl of a solution containing 0.2 M KCl, 20 mM Hepes pH 7.4, 2 mM DTT, 100 μM Ca^2+^ and a mixture of protease inhibitors: aprotinin (76.8 nM), benzamidine (0.83 mM), iodoacetamide (1 mM), leupeptin (1.1 mM), pepstatin (0.7 mM) and PMSF (0.1 mM), in the presence of the indicated concentrations of Sorcin. The bound [3H]ryanodine was separated from free ligand by filtering through Whatman GF/B glass fiber microfilters. The filters were washed with ice-cold water. Radioactivity remaining in the filters was measured by liquid scintillation counting. Specific binding was calculated as the difference between total and nonspecific binding measured in parallel assays in the presence of 20 μM unlabeled ryanodine. All experiments were performed in triplicate.

### Surface plasmon resonance (SPR) experiments

SPR experiments were carried out using a SensiQ Pioneer system (ICx Nomadics), essentially as in Genovese et al.^46^. The sensor chip (COOH5) was activated chemically by a 35 μl injection of a 1:1 mixture of N-ethyl-N′-(3-(diethylaminopropyl)carbodiimide (200 mM) and N-hydroxysuccinimide (50 mM) at a flow rate of 5 μl/min.

For FastStep experiments, the ligand, i.e., human Sorcin, was immobilized on activated sensor chips via amine coupling. The immobilizations were carried out in 10 mM sodium acetate at pH 4.0; the remaining unreacted groups were blocked by injecting 1 M ethanolamine hydrochloride (35 μl). The immobilization level of Sorcin was 2000 RU.

Analytes were RyR2 peptides corresponding to calmodulin-binding sites CaMBD1 (FRYNEVMQALNMSAALTARKTREFR), CaMBD2 (RSKKAVWHKLLSKRKRAVVACFRMAP), and CaMBD3 (ALRYNVLTLVRMLSLKSLKKQMKRMKKMTVK)^48^, synthesized by Biomatik (purity > 90%).

The analytes were dissolved in buffer 20 mM Hepes pH 7.4, 150 mM NaCl + 0.005% surfactant P20 (HBS-P buffer) (±100 μM CaCl_2_) to a concentration of 100 μM, automatically further diluted in HBS-P and injected on the sensor chip using FastStep assays, at the following concentrations: 1.56 μM (1–30 s), 3.125 μM (31–60 s), 6.25 μM (61–90 s), 12.5 μM (91–120 s), 25 μM (121–150 s), 50 μM (151–180 s) and 100 μM (181–195 s) at a constant flow (nominal flow rate = 100 μl/min, step contact time = 30 s), both in the presence of 1 mM EDTA and in the presence of CaCl_2_ at 100 μM concentration. The increase in RU relative to baseline indicates complex formation; the plateau region represents the steady-state phase of the interaction (RUeq), whereas the decrease in RU after 195 s represents dissociation of peptides from immobilized Sorcin after injection of buffer. Control experiments were performed in sensor chips treated as described above, in the absence of immobilized ligand. Regeneration procedures are based on two long (2000 s and 500 s) injections of buffer, separated by a brief (5 s) injection of 10 mM NaOH. Kinetic evaluation of the sensorgrams was obtained using the SensiQ Qdat program and full fitting with 1, 2, and 3 sites.

For OneStep SPR experiments, the ligands, i.e., GST-Sigma-1 receptor and GST (the control ligand) were immobilized on activated sensor chips via amine coupling. The immobilizations were carried out in 10 mM sodium acetate at pH 4.2; the remaining unreacted groups were blocked by injecting 1 M ethanolamine hydrochloride (35 μl). The immobilization level of GST was 80 RU, while the immobilization level of GST-Sigma-1 receptor was 125 RU. The analyte, i.e. human Sorcin, was dialysed in buffer 20 mM Hepes pH 7.4, 150 mM NaCl + 0.005% surfactant P20 (HBS-P) + 100 μM CaCl_2_ (HBS-PC buffer) or +1 mM EDTA (HBS-PE buffer), and injected on the sensor chip using OneStep assay as in Genovese et al.^46^, where Taylor dispersions were exploited to generate analyte concentration gradients that provide high-resolution dose-response in single injections. Full analyte titrations were recorded over four orders of magnitude in concentration, up to 5 μM. The increase in RU relative to baseline indicates complex formation; the plateau region represents the steady-state phase of the interaction (RUeq), whereas the decrease in RU represents dissociation of analytes from immobilized ligands after injection of buffer. The OneStep experiments in Figs. 4 and S7 are the difference between the interaction of Sorcin with GST-Sigma-1 receptor, and that with GST alone as a control. Kinetic evaluation of the sensorgrams was obtained using the SensiQ Qdat program and full fitting with 1 site.

### Cell Lines

HeLa and SHSY-5Y cells (ATCC, tested for mycoplasma contamination) were grown in a 5% CO_2_ atmosphere in Dulbecco’s modified Eagle’s medium high glucose (DMEM; Euroclone), supplemented with 10% fetal bovine serum (Gibco), 100 U/ml penicillin and 100 mg/ml streptomycin. Mock cells were maintained in growth medium, which was changed simultaneously with the beginning of treatments.

### Vectors and transfection

Twelve hours before transfection, HeLa cells were seeded onto 13 mm glass coverslips and allowed to grow to 50% confluence. Cells were transfected by calcium phosphate^49^. SPLICS constructs were obtained and used as in Cieri et al.^50^. For co-transfection, the two SPLICS ER and mitochondrial fragments were in a 1.5:2 ratio with the overexpressed protein of interest.

### Immunocytochemistry

Transfected cells overexpressing the protein of interest, were plated on 13 mm glass coverslips and fixed 48–72 h post transfection with 3.7% formaldehyde in phosphate-buffered saline (PBS; 140 mM NaCl, 2 mM KCl, 1.5 mM KH_2_PO_4_, 8 mM Na_2_HPO_4_, pH 7.4) for 20 min and washed three times with PBS. Cell permeabilization was performed by 20 min incubation in 0.1% Triton X-100/PBS followed by 30 min wash in 1% gelatin/PBS (type IV, from bovine skin, Sigma) and 15 min wash in PBS at room temperature (RT). The coverslips were then incubated for 90 min at 37 °C with the specific primary antibody diluted 1:20 in PBS. Further washing steps with gelatine and PBS were repeated as mentioned before to remove the excess of primary antibody. Staining was revealed by the incubation with specific AlexaFluor secondary antibodies (Thermo Fisher: Goat anti-Rabbit IgG AlexaFluor 405, Cat#A-31556; Goat anti-Rabbit IgG AlexaFluor 594, Cat#A-11012; Donkey anti-Goat IgG AlexaFluor 633, Cat#A-21082; Goat anti-Mouse IgG AlexaFluor 633, Cat#A-21050; Goat anti-Mouse IgG AlexaFluor 488, Cat#A-11001; Goat anti-Rabbit IgG AlexaFluor 488, Cat#A-11008) for 45 min at RT (1:50 dilution in PBS; 1:20 only for Goat anti-Rabbit IgG AlexaFluor 405). After further washing steps, coverslips were mounted using Mowiol 4-88 (Sigma). The coverslips were observed at the SP5 Leica confocal microscope at lasers wavelength of 405, 458, 488, 543, 555, and 633 nm.

### Subjects

All the human brain samples were obtained from the University of California-Irvine-ADRC Brain Tissue Repository, the Eunice Kennedy Shriver NICHD Brain and Tissue Bank for Developmental Disorders, and the University of Kentucky ADC. Down syndrome (DS) cases were divided into two groups, with or without sufficient pathology for a neuropathology diagnosis of AD. The age of DS cases is under 40, while the cases with both DS and AD (DSAD) were over the age of 40 years. Likewise, controls were split into two groups: Controls Young (Ctr Y), which match with DS because they are ≤45 years; Controls Old (Ctr O), which match with DS/AD because they are older than 45 years at death.

All the results obtained from human autopsy samples were analyzed by considering the difference in postmortem interval (PMI) among groups.

*Substantia nigra* from R6/2 mice and from control mice was obtained from Neuromed Laboratories.

### Sample preparation and western blot analysis

In all experiments, total protein extracts were prepared in RIPA buffer (pH 7.4) containing Tris-HCl (50 mM, pH 7.4), NaCl (150 mM), 1% NP-40, 0.25% sodium deoxycholate, EDTA (1 mM), 0.1% sodium dodecyl sulfate (SDS), supplemented with proteases inhibitors [phenylmethylsulfonyl fluoride (PMSF, 1 mM), sodium fluoride (NaF, 1 mM) and sodium orthovanadate (Na_3_VO_4_, 1 mM)].

Cell extracts and cortex samples from HD and DS brain and control were homogenized by 20 passes with a Wheaton tissue homogenizer and centrifuged for 1 h at 16,000 × *g*, 4 °C to remove cellular debris.

The supernatant was then extracted to determine the total protein concentration by the bicinchoninic acid assay (Pierce, Rockford, IL., USA).

For western blots, 20 μg (30 μg for experiments in Fig. 6) of proteins were resolved on 12% SDS-PAGE using Criterion Gel TGX and blotted onto a nitrocellulose membrane (Bio-Rad, Hercules, Calif., USA). Before the transfer process, the image of the total protein load was acquired to perform normalization of blot data analysis. The membranes were blocked with 3% bovine serum albumin in 0.5% Tween-20/Tris buffered saline (TTBS) and incubated overnight at 4 °C with primary antibodies: Anti-Sorcin (home made^13^ or Abcam Ab71983). After 3 washes with TTBS the membranes were incubated for 60 min at room temperature with anti-rabbit IgG secondary antibody conjugated with horseradish peroxidase (1:5000; Sigma-Aldrich, St Louis, MO, USA). Membranes were developed with the Super Signal West Pico chemiluminescent substrate (Thermo Scientific, Waltham, MA, USA), acquired with Chemi-Doc MP (Bio-Rad, Hercules, CA, USA) and analyzed using Image Lab software (Bio-Rad, Hercules, CA, USA) that permits the normalization of a specific protein signal with the β-actin signal in the same lane or total proteins load. β-actin was evaluated using primary antibody (1:1000, Cell Signaling β-Actin Mouse mAb #3700), and anti-mouse IgG secondary antibody conjugated with horseradish peroxidase (Sigma-Aldrich, St Louis, MO, USA).

### Ca^2+^ and ER-mitochondria contact sites measurements

Ca^2+^ measurements were performed by co-transfecting HeLa cells in a six-well plate with cytosolic (cytAEQ) and ER-targeted (erAEQ) aequorin along with either empty pcDNA3 vector or with Sorcin expression vector in a 1:2 ratio favouring Sorcin. Forty-eight hours post transfection, cells were re-plated into a 96-wells plate (PerkinElmer). CytAEQ was reconstituted by incubating cells for 1.5 h with 5 µM coelenterazine wt (Santa Cruz Biotech) in modified Krebs Ringer Buffer (KRB: 125 mM NaCl, 5 mM KCl, 400 mM KH_2_PO_4_, 1 mM MgSO_4_, 20 mM Hepes, pH 7.4) supplemented with 5 mM glucose at 37 °C. Luminescence measurements were carried out using a PerkinElmer EnVision plate reader equipped with two injector units. After reconstitution, cells were placed in 70 µl of KRB solution and luminescence from each well was measured for 1 min. During the experiment, 100 µM histamine at the final concentration were first injected to activate Ca^2+^ transients, and then a hypotonic, Ca^2+^-rich, digitonin-containing solution was added to discharge the remaining aequorin pool. ErAEQ was reconstituted by incubating cells for 1 h with 5 µM coelenterazine n (Santa Cruz Biotech) in modified Krebs Ringer Buffer (KRB: 125 mM NaCl, 5 mM KCl, 400 mM KH_2_PO_4_, 1 mM MgSO_4_, 20 mM Hepes, pH 7.4) supplemented with 5 mM glucose, 5 µM ionomycin and 600 μM EGTA at 4 °C. ER-mitochondria contact sites were assessed by co-transfecting cells with SPLICS and either empty vector or Sorcin expression vector in a ratio 1:2. Forty-eight hours post transfection cells were fixed and imaged as already described^49^. Output data were analyzed and calibrated with a custom-made macro-enabled Excel workbook.

### Proximity ligation assay

HeLa cells were transfected with Lipofectamine 2000 or RNAi MAX (ThermoFisher Scientifics), respectively for Sorcin overexpression and silencing, according to the manufacturer’s instructions. 36 h after transfection, cells were washed in PBS, fixed for 10 min in 4% PFA at room temperature and, after PBS washing steps, permeabilized with a solution of PBS 0,1% Triton X-100 (v/v) for 10 minutes at room temperature. Cells were then blocked in PBS 5% BSA (w/v) solution for 1 h at room temperature and finally incubated overnight at 4 °C in a wet chamber with 1:100 dilution of anti-rabbit VDAC1 (Abcam Ab34726) and anti-mouse IP3R3 (BD Bioscience) antibodies.

PLA experiment was performed using PLA Duolink kit red fluorescent reagent and probes (Sigma-Aldrich) according to the manufacturer’s instruction and as described by Rimessi et al.^51^. The images were acquired with fluorescence microscope Zeiss Axiovert 200 M (×40 magnification) and analyzed with ImageJ.

### GST pulldown experiment

GST pulldown procedure was performed according to Genovese et al.^52^. Total protein content of SH-SY5Y cell lysate was quantified through Lowry quantification method (Thermo Scientifics) according to manufacturer’s instruction. 10 μg of cell lysate was loaded on SDS-PAGE as the input, while ~1 mg of the same extract was incubated with both GST-tagged Sorcin and GST, as negative control for binding, both in the presence of 500 μM CaCl_2_ and 1 mM EDTA. The pulled-down samples were then loaded on the same SDS-PAGE. Sorcin-GST (47 KDa) and GST tag (25 KDa) presence was checked through Ponceau-S staining, while the interactors were detected through western blot, using anti-mouse SERCA-2a antibody (Abcam, ab2861) and anti-rabbit Sigma-1R (SIGMA, HPA018002), both at a 1:1000 dilution.

### Image acquisition and processing

Cells were generally imaged 48–72 h after transfection with a Leica TSC SP5 inverted confocal microscope, using either a HCX PL APO 63X/numerical aperture 1.40–0.60 or a HCX PL APO × 100/numerical aperture 1.4 oil-immersion objective. Images were acquired by using the Leica AS software. To count ER-mitochondria contacts, a complete z-stack of the cell was acquired every 0.29 µm. Z-stacks were processed using Fiji:^49^ images were first convolved, and then filtered using the Gaussian Blur filter. A 3D reconstruction of the resulting image was obtained using the Volume J plugin (http://bij.isi.uu.nl/vr.htm). A selected face of the 3D rendering was then thresholded and used to count ER-mitochondria contact sites.

### Statistical analysis

Results shown are mean values ± SEM. Student’s unpaired two-tailed *t*-test was used for comparisons involving two groups when sample followed a Gaussian distribution, otherwise Mann–Whitney test was used. Differences between groups were considered significant when p ≤ 0.05. All statistical analyses were performed using GraphPad Prism version 6.00 for Mac OS X, GraphPad Software (La Jolla, California, USA). The exact values of *n* and their means are indicated in the figure legends. *p ≤ 0.05, **p ≤ 0.01.

## Results

### Sorcin expression and localization in the nuclear-perinuclear region, coimmunoprecipitation with RyR in neurons

We first studied expression and localization of Sorcin in cortex neurons and in other brain cell types ex vivo and in vitro.

**Fig. 2:**
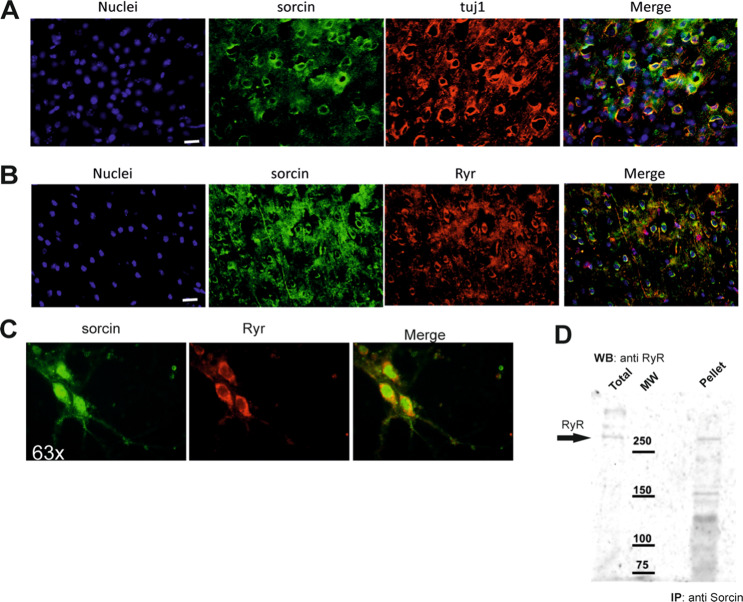
Sorcin partially colocalizes and co-immunoprecipitates with RyR. Immunofluorescence staining (**A**) for nuclei (blue), sorcin (green) and Tuj-1(red) and (**B**) for nuclei (blue), sorcin (green) and Ryr (red) on cortex region of adult mouse brain coronal sections. Scale bar: 20 μm. Immunofluorescence experiment showing mouse cortex neurons (division 8) stained with anti-Sorcin and mouse anti-RyR Magnification: ×63 (**C**). Sorcin partially colocalizes with RyR in all samples; Sorcin localized in perinuclear and in the axonal region. Immunoprecipitation experiments (**D**): Sorcin and RyR co-immunoprecipitate in the presence of calcium.

Sorcin is highly expressed in mouse cortex neurons and in microglia cells (Figs. 2A–C and S1, S2). Sorcin is localized mostly in the nuclear-perinuclear region of microglia (Fig. S2) and neurons (Figs. 2A, 2B and S1), where it partially colocalizes with ryanodine receptors, which are also highly expressed in cortical microglia and in cerebellar region (Figs. S4, S5). Further, Sorcin is also localized in the axonal region, where it partially colocalizes with beta-tubulin (Fig. S1) and in motor neuron-like cell line (Fig. S3).

Sorcin and RyR (located mainly in ER) co-immunoprecipitate from cortical neurons culture lysates, in the presence of CaCl_2_ at 100 μM concentration (Figs. 2D and S6), while no co-precipitation occurs in the presence of EDTA (data not shown).

### Sorcin inhibits RyR2 and RyR3 by interacting with calmodulin-binding domains

**Fig. 3:**
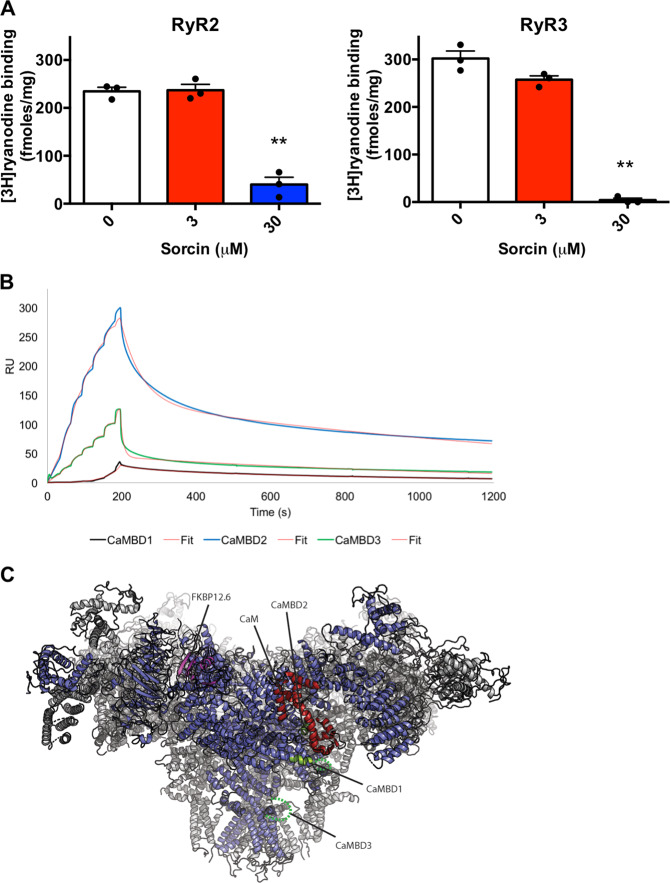
Sorcin inhibits [3H]ryanodine binding to RyR2 and to RyR3 and binds calmodulin-binding sites of RyRs with high affinity in vitro. **A** Sorcin inhibits [3H]ryanodine binding to both RyR2 and RyR3, highly expressed in the brain, in a concentration-dependent manner. **B** SPR FastStep titration experiments in the presence of 100 μM CaCl_2_ show that RyR2 calmodulin-binding sites CaMBD2 and CaMBD3 peptides bind with high affinity by Sorcin immobilized to a COOH5 sensorchip, while CaMBD1 binds Sorcin with lower affinity; best fits with full fittings with 2 sites (Sorcin is a homodimer). **C** Cryo-EM structure of mouse RyR2 at 3.6 A resolution (PDB ID 6JI8). One subunit is shown in blue, with the three others in gray. A bound apoCaM is shown in red, and FKBP12.6 in magenta. The positions of the three CaMBDs, used for interaction studies with Sorcin, are highlighted in green. CaMBD2 is directly bound to CaM. CaMBD1 is nearby, with part of it alpha-helical, and the remainder disordered (green dotted lines). CaMBD3 is in a disordered area (dotted lines). As such, all three CaMBDs would be accessible for binding Sorcin.

RyRs have different tissue specificities. Sorcin interaction and inhibition of RyR2 (expressed in cardiac muscle and brain, especially cerebellum) has already been described in cardiomyocytes^7–9^ and in caudate-putamen^27^; however, interaction of Sorcin with RyR3 (expressed in the corpus striatum, in brain cortex, and in the smooth muscle) has never been studies to date. Microsomes from RyR2 and RyR3-expressing HEK293 cells vs. control were incubated with 9 nM [3H]ryanodine, that typically increases channel conductance^53^, in a solution containing 0.2 M KCl and different Sorcin concentrations: Sorcin inhibits [3H]ryanodine binding to both RyR2 and RyR3, highly expressed in the brain, in a concentration-dependent manner (Fig. 3A), thereby inhibiting both RyR2 and RyR3.

The molecular basis of the inhibition of RyR2 and RyR3 by Sorcin was studied by SPR studies, where the binding of Sorcin to RyR regions already demonstrated to be important for calmodulin-dependent regulation has been investigated^48^. We studied the calcium-dependent binding of Sorcin to 3 RyR calmodulin-binding peptides: in the presence of 100 μM CaCl_2_ Sorcin interacts with CaMBD2 (calmodulin-binding domain 2) and with CaMBD3 in the submicromolar range (*K*_D CaMBD2_ = 120 nM; *K*_D CaMBD3_ = 480 nM), while interaction with CaMBD1 occurs with *K*_D_ values above 10 μM (Fig. 3B). Sorcin interacts with RyR in the same sites, and with affinities in the same order CaMBD2 > CaMBD3 > CaMBD1 observed for calmodulin^48^. In the absence of calcium, the interaction between Sorcin and CaMBDs is negligible (data not shown), indicating the ability of Sorcin to bind RyRs when the channels are open (high local cytosolic calcium concentration), and to regulate them, determining their closure, and consequently a decrease of cytosolic calcium concentration.

The CaMBDs are located in the cytosolic cap of the RyRs, according to a recent RyR2-calmodulin cryo-EM structure (Fig. 3C), where they are exposed to interactions with regulating proteins; the CaMBDs, and especially CaMBD2, are important for RyR regulation, and can be therefore bound and regulated by Sorcin^48^.

### Sorcin interacts in a calcium-dependent fashion with SERCA2 and the Sigma-1 receptor

In addition to Sorcin-RyR2 and -RyR3 interaction, we studied whether Sorcin is able to interact in a calcium-dependent fashion with proteins important for calcium dysregulation, ER and mitochondrial stress in pathological conditions. We, therefore, studied the interaction of Sorcin with other most important ER calcium metabolism proteins, i.e., SERCA (the ATP-dependent calcium pump responsible for ER calcium filling, able to pump Ca^2+^ from the cytosol of the cell to the lumen of the ER) and the Sigma-1 receptor, a chaperone protein at the endoplasmic reticulum (ER) that modulates calcium signaling through the IP3 receptor.

**Fig. 4:**
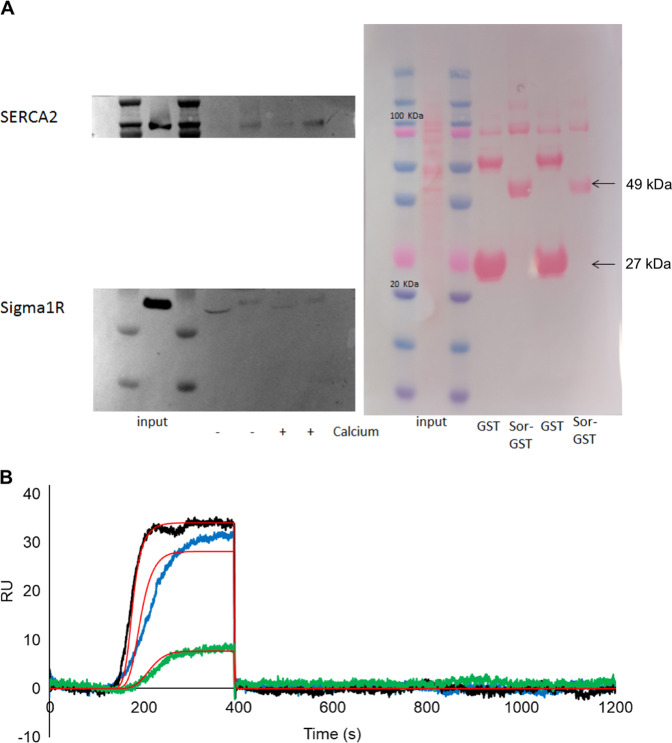
Sorcin interacts with SERCA2 and the Sigma-1 receptor. **A** In the presence of 500 μM CaCl_2_ (+ Calcium), Sorcin interacts with SERCA2 and (less specifically) with the Sigma-1 receptor in SH-SY5Y cells. In the presence of 1 mM EDTA (− Calcium), decreased interaction can be observed. **B** OneStep SPR experiment showing the interaction of Sigma-1 receptor (1–138) immobilized on a COOH5 chip with human Sorcin (analyte) in the presence of 500 μM CaCl_2_. In OneStep assays, Taylor dispersions were exploited to generate analyte concentration gradients that provide high-resolution dose-response in single injections. Full analyte titrations were recorded over four orders of magnitude in concentration, up to 5 μM. The experiment are titrations of Sorcin at concentrations of 200 nM (green), 1.2 μM (blue) and 5 μM (black). The increase in RU relative to baseline indicates complex formation between Sorcin and Sigma-1 receptor; the plateau region represents the steady-state phase of the interaction (RUeq), whereas the decrease in RU represents dissociation of analyte from immobilized ligand after injection of buffer. Kinetic evaluation of the sensorgrams (red lines) was obtained using the SensiQ Qdat program and full fitting with 1 site.

GST pulldown experiments were performed in SH-SY5Y cells (Fig. 4A). In the presence of 500 μM CaCl_2_, Sorcin was found to interact with SERCA2 and (less specifically) with the Sigma-1 receptor in SH-SY5Y cells. In the presence of 1 mM EDTA, decreased interaction could be observed. OneStep SPR experiments were carried out to measure the interaction between immobilized Sigma-1 receptor and Sorcin, in the presence of 500 μM calcium or in the presence of 1 mM EDTA: in the presence of calcium, a *K*_D_ of 720 nM was calculated, while in the presence of EDTA a *K*_D_ of 12 μM was obtained (Figs. 4B and S7).

### Sorcin expression in models of neurodegenerative diseases

Sorcin expression was evaluated in cellular, animal and patient models of neurodegenerative diseases, to evaluate whether it may represent a marker of neurodegeneration, by western blot experiments.

**Fig. 5:**
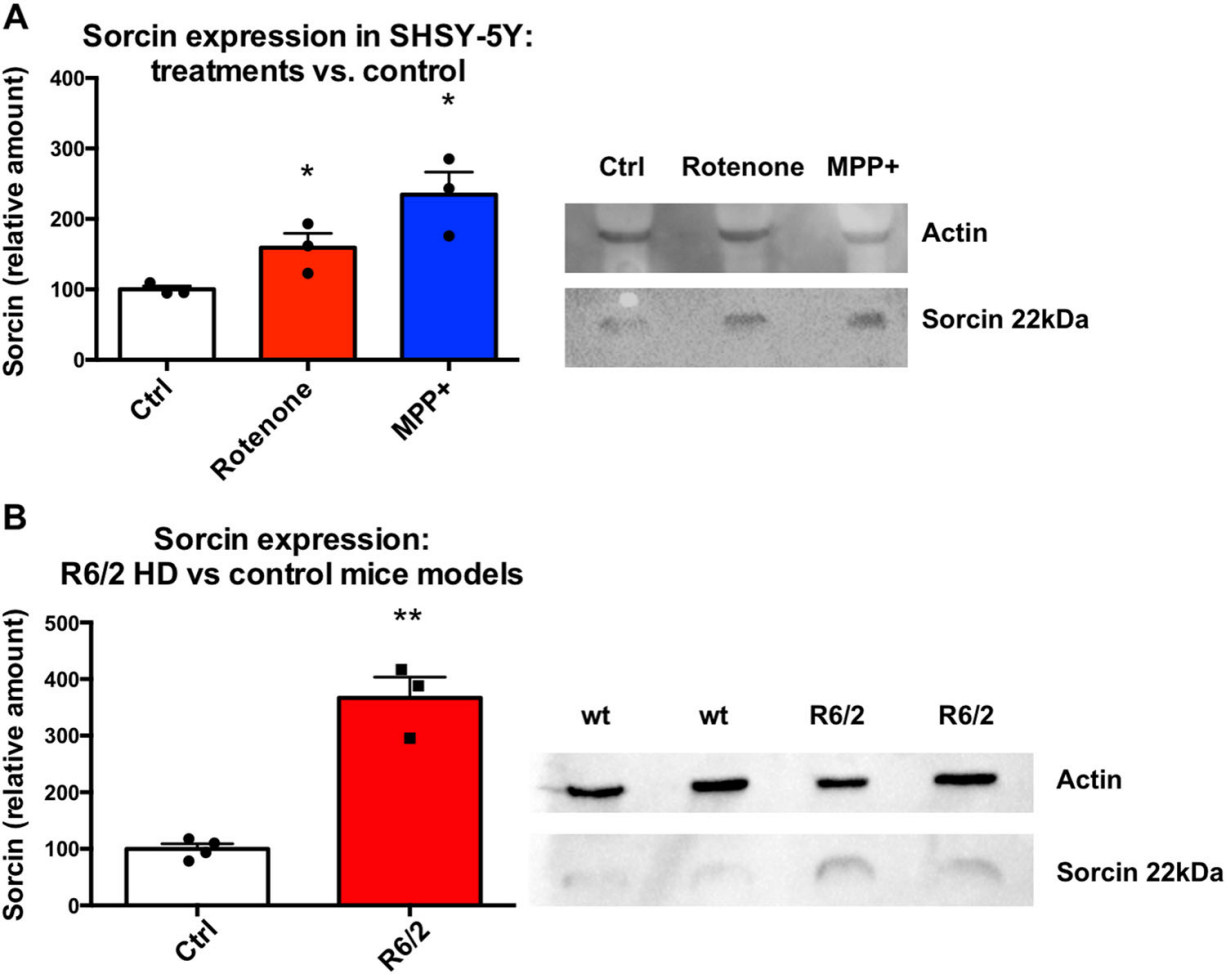
Sorcin expression in neurodegenerative diseases models. **A** Sorcin expression levels in SH-SY5Y cells upon 48 h treatment with MPP+ and Rotenone, vs. control cells. Cells treated with MPP+ (1 μM) and cells treated with Rotenone (100 nM) express increased amount of Sorcin with respect to control cells (*p* < 0.05). The experiment carried out in triplicate; mean ± SEM is shown. On the right, representative Western blot (see also Fig. S8). **B** Sorcin expression levels in *substantia nigra* of murine model of HD vs. control mice. *Substantia nigra* from R6/2 mice express high amount of Sorcin with respect to control (*p* < 0.01). The experiment carried out in triplicate; mean ± SEM is shown. On the right, representative western blot (see also Fig. S9).

Sorcin expression levels were measured in SH-SY5Y cells upon 48 h treatment with MPP^+^ (1-methyl-4-phenylpyridinium) and Rotenone, vs. control cells (Figs. 5A and S8), to test whether Sorcin expression is increased in conditions of mitochondrial oxidizing stress. MPP^+^ is a positively charged organic neurotoxin widely used in animal models of PD, that acts by interfering with oxidative phosphorylation in mitochondria by inhibiting complex I, by promoting the formation of reactive free radicals in the mitochondria of dopaminergic neurons, with overall inhibition of the electron transport chain eventually leading to the depletion of ATP and eventual cell death, especially of the dopaminergic neurons. Cells treated with MPP^+^ (1 mM) express 235 ± 46% Sorcin with respect to control (*p* = 0.014). Rotenone is a toxin that interferes with the electron transport chain in mitochondria, inhibiting the transfer of electrons from iron-sulfur centres in complex I to ubiquinone. This interferes with NADH during the creation of ATP. Complex I is unable to pass off its electron to CoQ, creating a back-up of electrons within the mitochondrial matrix. Cellular oxygen is reduced to the radical, creating reactive oxygen species, which can damage DNA and other components of the mitochondria; rotenone also inhibits microtubule assembly. Cells treated with Rotenone (100 nM) express 159 ± 30% Sorcin with respect to control cells (*p* = 0.042).

Since Sorcin is known to be overexpressed in *substantia nigra* of PD patients^39,40^, and the release of high amount of dopamine from *substantia nigra* pars compacta is one of the most important early features of HD^54^, we explored the expression of Sorcin in the same brain region of the most commonly used murine model of HD (caused by an autosomal dominant CAG expansion mutation of the huntingtin gene) vs. control mice. R6/2 mice model human HD by expressing a portion of the human huntingtin (*Htt*) gene under human gene promoter elements (1 kb of 5 UTR sequence and exon 1 together with ~140 CAG repeats). Expression of this amino-terminal fragment of the huntingtin protein with its polyglutamine expansion is sufficient to reproduce the phenotype of human HD. *Substantia nigra* from R6/2 mice has increased expression (367 ± 36%) of Sorcin with respect to control (*p* = 0.007) (Figs. 5B and S9).

**Fig. 6:**
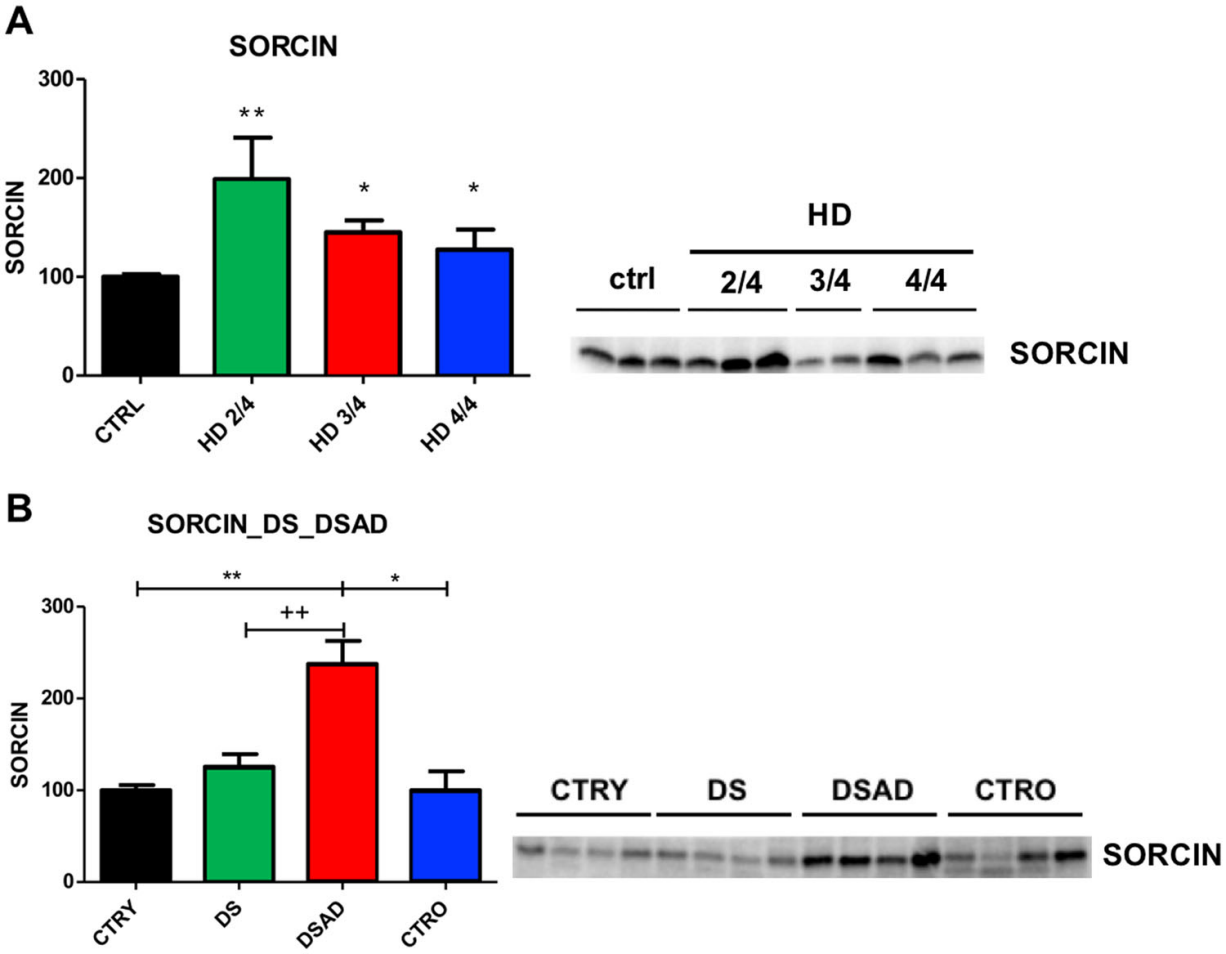
Sorcin expression in neurodegenerative disease patients. **A** Sorcin expression (averages, standard deviations) in HD patients: relative Sorcin expression in samples from controls (CTRL), patients with Huntington’s disease stage 2/4, 3/4, 4/4. Patients with HD stage 2/4 express significantly higher Sorcin than control patients (*p* < 0.01) and patients with HD stage 3/4 and 4/4 (*p* < 0.05). Mean ± SEM is shown. A representative western blot is shown (see also Fig. S10). **B** Sorcin expression (averages, standard deviations) from experiments in Fig. S11: relative Sorcin expression in samples from controls (CTRY, CTRO), patients with Down syndrome (DS), patients with Down syndrome and with Alzheimer’s Disease (DSAD). DSAD patients have statistically significantly higher Sorcin expression than in controls and DS patients. Mean ± SEM is shown. A representative western blot is shown.

Sorcin expression is increased significantly in cortex of HD patients stage 2/4, 3/4 and 4/4 of disease progression with respect to control cortices (Figs. 6A and S10); Sorcin is particularly upregulated (+99 ± 42%, *p* < 0.01) in patients at stage 2/4 (early intermediate stage), almost fully functional, indicating that it may represent an early marker of neurodegeneration.

Sorcin expression was also assessed in cortex samples from young and old controls (CTRY, CTRO), patients with Down syndrome (DS) and patients with Down syndrome and with Alzheimer’s Disease (DSAD). DSAD patients have statistically significantly higher Sorcin expression than in controls and DS patients (Figs. 6B, S11).

### Sorcin expression affects the Ca^2+^ uptake rate in the ER

**Fig. 7:**
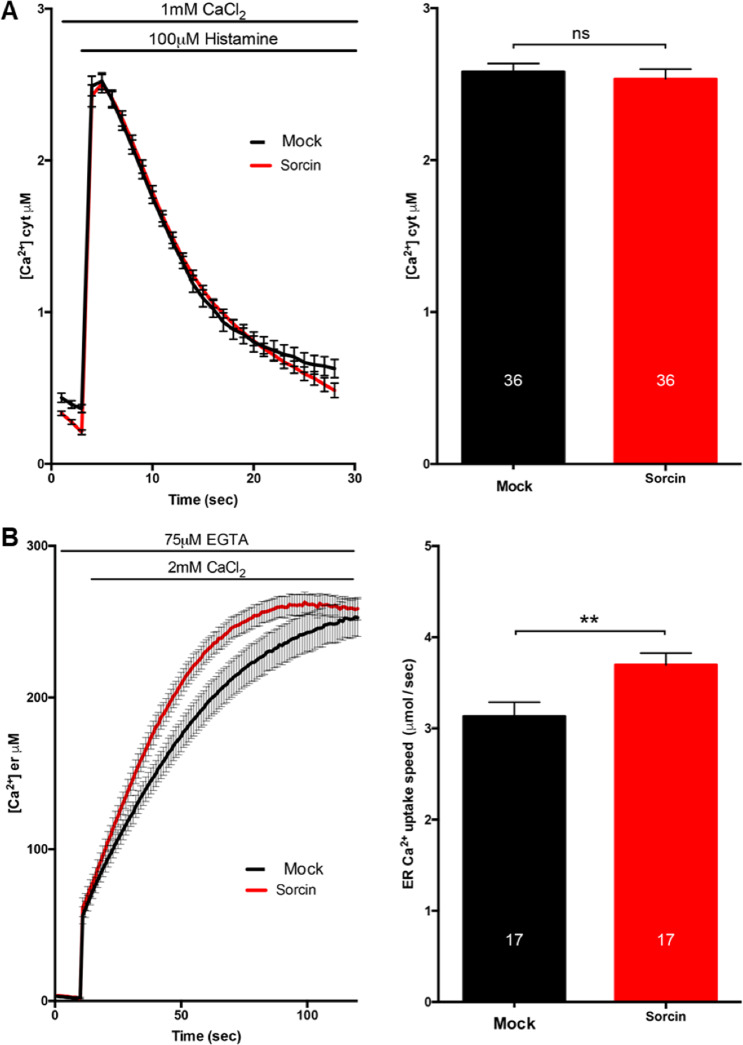
Overexpression of Sorcin enhances the velocity of ER Ca^2+^ transients in HeLa cells. **A** Cytosolic [Ca^2+^]_c_ and **B** the kinetics of ER refilling upon re-addition of CaCl_2_ 1 mM to Ca^2+^-depleted cells in HeLa cells either mock transfected or overexpressing Sorcin are shown. Cells were transfected with AEQ (either cytosolic or targeted to the ER) (controls, cytAEQ, or erAEQ, respectively) or co-transfected with AEQ and Sorcin. Traces refer to representative experiments selected from at least three independent experiments. Quantification of [Ca^2+^]_c_ and speed uptake of [Ca^2+^]_er_ in HeLa cells mock transfected or overexpressing Sorcin is also shown.

**Fig. 8:**
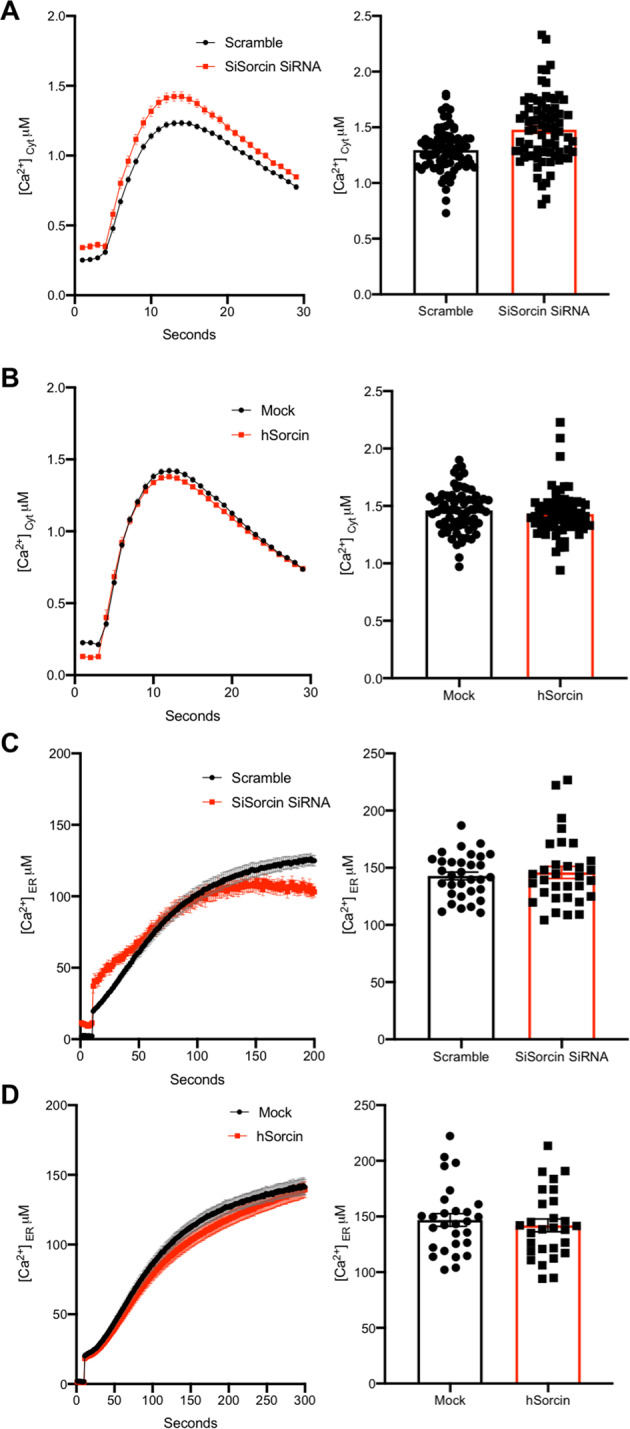
Sorcin silencing increases cytosolic calcium and decreases the velocity of ER Ca^2+^ transients in SH-SY5Y cells. Cytosolic [Ca^2+^]_c_ upon re-addition of CaCl_2_ 1 mM to Ca^2+^-depleted cells in SH-SY5Y cells upon addition of Sorcin-silencing (red) vs. scramble (black) siRNA (**A**), or upon mock transfection (black) or overexpressing Sorcin (red) (**B**) are shown. The kinetics of ER refilling upon re-addition of CaCl_2_ 1 mM to Ca^2+^-depleted cells in SH-SY5Y cells upon addition of Sorcin-silencing (red) vs. scramble (black) siRNA (**C**), or upon mock transfection (black) or overexpressing Sorcin (red) (**D**) are also shown. Cells were transfected with AEQ (either cytosolic or targeted to the ER) (controls, cytAEQ, or erAEQ, respectively) or co-transfected with AEQ and Sorcin. Traces refer to representative experiments selected from at least three independent experiments. Quantification of [Ca^2+^]_c_ and speed uptake of [Ca^2+^]_er_ in SH-SY5Y cells Sorcin-silencing vs. scramble siRNA, or upon mock transfected (black) or overexpressing Sorcin (red) is also shown.

Perturbed ER calcium homeostasis, ER stress, altered cytosolic calcium buffering are early hallmarks of neurodegeneration. Since Sorcin is able to interact and regulate important players in these events, and it is highly expressed in brain, and further overexpressed in neurodegenerative diseases models, especially as an early stress marker, luminescence-based measurements of cytosolic and ER calcium transients were carried out both in HeLa cells and in a brain-derived tumor cell line (SH-SY5Y) to evaluate Sorcin-dependent Ca^2+^ cellular regulations. We thus performed Ca^2+^ measurements using organelle-targeted aequorin probes specific for the cytoplasm (cytAEQ) or the ER lumen (erAEQ)^49^, both in HeLa cells (Fig. 7) and in SH-SY5Y cells (Fig. 8).

Cytosolic Ca^2+^ transients of HeLa cells overexpressing Sorcin compared to the control transfected cells when exposed to the InsP_3_-linked agonist histamine (100 µM; Fig. 7A) was substantially unaltered. ER Ca^2+^ transients were slightly altered; the calcium uptake rate in the ER was instead increased by about 24% in Sorcin-overexpressing HeLa cells with respect to controls (Fig. 7B), possibly because of SERCA^19^. Sorcin therefore regulates ER calcium fluxes, increasing calcium entry in the organelle.

In SH-SY5Y cells, we measured cytosolic and ER calcium transients both in Sorcin-silenced vs. scrambled siRNA-treated cells, and in Sorcin-overexpressing vs. mock cells (Fig. 8). In Sorcin-silenced SH-SY5Y cells, the cytosolic transients result increased in cells, while the calcium uptake rate in the ER is slower than in the control.

Overall, Sorcin overexpression tends to increase ER SERCA-dependent calcium uptake rate. On the contrary, Sorcin silencing decreases ER SERCA-dependent calcium uptake rate, increasing the cytosolic calcium: these represent early markers of neurodegeneration. Increased Sorcin expression in ER-stressing condition may counterbalance such stress.

### Sorcin overexpression alters ER-mitochondria contacts (MAMs)

**Fig. 9:**
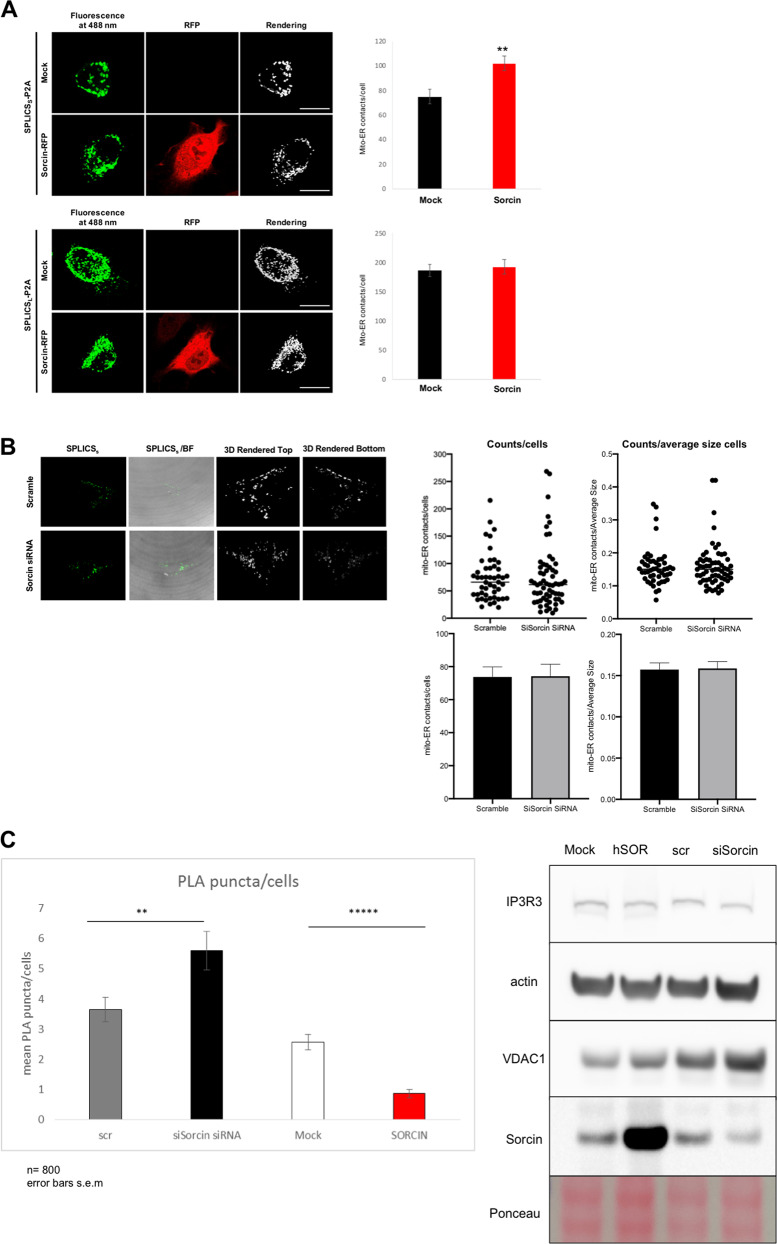
Sorcin overexpression alters ER-mitochondria contacts (MAMs). **A** Effect of Sorcin overexpression on mitochondrial-ER contact sites: HeLa cells upon mock transfection (black) or overexpressing Sorcin (red), were co-transfected with Sorcin-RFP expression vectors and the short-range (top) and long-range (bottom) SPLICS sensor to assess ER-mitochondria associations. Reconstitution of the fluorescent signal was observed upon 488 nm wavelength excitation in Sorcin positive cells. The 3D rendering of the Z-stacks acquired for the SPLICS probes is shown on the right. Quantification of the ER-mitochondria contact sites/cell in the different conditions is shown as mean ± SEM. Scale bar 20 µm. **B** Effect of Sorcin silencing on ER-mitochondria contact sites: HeLa cells upon addition of Sorcin-silencing vs. scramble siRNA were co-transfected with Sorcin-RFP expression vectors and the short-range (top) and long-range (bottom) SPLICS sensor to assess ER-mitochondria associations. Reconstitution of the fluorescent signal at 488 nm wavelength excitation in Sorcin positive cells and the 3D rendering of the Z-stacks acquired for the SPLICS probes are shown in Fig. S13. **C** Left: PLA experiments showing the alteration of IP3R3-VDAC1 interaction in HeLa cells upon addition of scramble siRNA (gray) vs. Sorcin-silencing siRNA (black), upon mock transfection (white) vs. overexpressing Sorcin (red). Right: alteration of IP3R3 and VDAC expression in representative western blot experiments is shown.

We have then evaluated the effect of Sorcin expression on MAMs in HeLa cells, using split-GFP-based contact site sensor (SPLICS) engineered to fluoresce when organelles are in proximity^50^, and a PLA-based approach, to assess whether Sorcin overexpression, observed in neurodegenerative diseases models, not only alters ER calcium handling, but also affects ER-mitochondrial contacts (Fig. 9). Indeed, the two techniques aim at different scopes; in one case PLA gives an idea of the proximity of two proteins involved in functional Ca^2+^ transfer between the organelles, while SPLICS-based approach evaluates the juxtaposition of ER and mitochondria, giving mostly a structural information that can be suggestive of a functional readout in calcium signaling.

By using the short-range ER-mitochondria interactions sensor (SPLICS_S_), Sorcin overexpression in HeLa cells was able to increase the number of narrow juxtapositions between ER and mitochondria (8–10 nm), i.e., the range in which an efficient Ca^2+^ transfer between the two organelles can take place, by about 36% (*p* < 0.01) (Fig. 9A, top), while the number of long SPLICS (SPLICS_L_), measuring wide juxtapositions between ER and mitochondria (40–50 nm) is not significantly altered by Sorcin overexpression (Fig. 9A, bottom), suggesting the possibility that Sorcin might act specifically at the organelle interface to selectively fine-tune Ca^2+^-dependent signals. Sorcin silencing does not alter substantially the number of contacts, nor the count/average size cells (Fig. 9B).

In situ proximity ligation assay (PLA) experiments were also performed, where close proximity between proteins of the outer mitochondrial membrane (VDAC) and of the ER membrane (IP3R3) at the MAM interface was studied following Sorcin overexpression or silencing in HeLa cells (Fig. 9C). IP3R3-VDAC1 tethering system is one of the functional junctions between ER and mitochondria to permit the calcium transfer at MAMs^51,55,56^. The mean number of PLA puncta per cells results increased by silencing Sorcin and decreased in Sorcin-overexpressing cells. This result, apparently in contrast with that obtained with SPLICS-based approach, can be explained with the fact that Sorcin expression might affect “structurally” the formation of IP3R3-VDAC1 tethering system, while on the other hand still facilitating the proximity of the two organelles via different tethering systems.

Overall, the early effect of Sorcin overexpression in ER stress conditions alters ER-mitochondria contacts in a complex fashion: we can only hypothesize that interaction of Sorcin with MAM proteins (and possibly their regulation) might explain the changes at mito-ER contact sites.

## Discussion

AD, PD and HD, despite affecting different brain regions in different fashions, share several features; Ca^2+^ dysregulation and ER stress are early hallmarks of neurodegeneration^2,3,57^. Calcium dysregulation contributes to the characteristic features of neurodegenerative diseases and, conversely, neurodegeneration induced by amyloid-β or Tau, by mHtt and by αSyn is at least partially mediated by altered calcium homeostasis. Perturbed endoplasmic reticulum (ER) calcium homeostasis, ER stress, and the consequent production of unfolded proteins are involved in the accumulation and deposits of misfolded proteins in neurodegenerative diseases. Calcium is crucial to normal brain function and, accordingly, its role in disease is likely to involve disruption of multiple pathways; dysregulation of [Ca^2+^]_i_ is a common event leading to the decline in the normal neuronal functions and eventually its death. Ca^2+^ can affect Tau phosphorylation, APP processing, and lysosome function. Disruption in any or all of these Ca^2+^-dependent cellular mechanisms can lead to catastrophic results for the brain, including aberrant neural network activity, synapse degeneration and cell death, and consequent cognitive deficits.

Brain regions are able to upregulate calcium buffering, indicating that they can adapt to calcium dysregulation. Many actors play a role in these processes. Signal transduction via Ca^2+^/calmodulin is linked to numerous major cellular functions of neurons including activating calcium-dependent kinases (e.g., CaMKII, PKC) or phosphatases, regulating intrinsic excitability, pre-/and post-synaptic plasticity, nitric oxide production, mitochondrial function, and cellular bioenergetics.

Sorcin: (i) is an essential protein, one of the most expressed calcium-binding proteins in the human brain; (ii) is overexpressed in models of neurodegenerative diseases; (iii) regulates calcium homeostasis by regulating calcium channels (RyR and SERCA in the ER), increases ER Ca^2+^ load, participates in preventing ER stress and the unfolded protein response; iv) interacts in a calcium-dependent fashion with αSyn, presenilin2 (PS2) and other proteins, and regulates PS2^58,59^; for these reasons, Sorcin may represent a useful marker and an important player in the mechanisms of neurodegeneration.

In the present work, we demonstrate that Sorcin is overexpressed in several models of neurodegenerative diseases, ranging from cellular models of PD to murine models of HD, to HD and AD patients.

In neuronal cells, Sorcin partially interacts with RyRs, possibly by interacting with the regulating N-terminal domains of the channel, also regulated by calmodulin. Sorcin is able to decrease RyR-dependent calcium efflux from the ER. The only way to discriminate between RyR2 and RyR3 is to use artificial systems, as reconstituted microsomes with overexpressed RyR2 or RyR3, in order to measure RyR activity (in this case, ryanodine binding as a function of Sorcin concentration): we demonstrate that Sorcin regulates not only RyR2, but also the other brain-specific RyR3 channel, by interacting with the very same sites regulated by calmodulin, in particular CaMBD2.

We also demonstrate that Sorcin interacts in a calcium-dependent fashion with Sigma-1 receptor, another protein that functions at the ER to regulate calcium signaling, bioenergetics, and ER stress^44^.

In conditions that determine ER stress, Sorcin, which is expressed at rather high levels in the brain, is further upregulated, to maintain low cytosolic and high ER calcium levels, and to decrease ER stress. Sorcin expression is possibly a first form of defence used by the cells (neurons, in particular) to counteract ER stress; this could be the cause for the overexpression of Sorcin in HD 2/4 stage, higher than in the more advanced 3/4 and 4/4 stages.

Often neuronal cells have dysregulated Ca^2+^ homeostasis and express low concentration of calcium buffering proteins, when aging and/or hit by neurodegenerative diseases and are more prone to undergo ER stress^60,61^; in turn, Sorcin is highly expressed in the brain, and is further overexpressed in human, mice and cellular neurodegenerative diseases models, and is, therefore, an important player in the calcium-linked mechanisms at the basis of neurodegeneration.

Alterations of calcium fluxes have complex, concentration-dependent and time-dependent effects on mitochondrial bioenergetics^62^. We demonstrate that Sorcin controls Ca^2+^ flux across cell compartments to counteract Ca^2+^ unbalance caused by neurodegeneration; in particular, Sorcin is overexpressed under ER stress; Sorcin interacts with proteins involved in ER homeostasis, as Ryr2, RyR3, SERCA, and Sigma-1 receptor, increases ER calcium transients, implying complex metabolic disturbances that are early events of neurodegeneration^63^.

In addition, we studied the effect of Sorcin on MAMs, using two different techniques: PLA gives an idea of the proximity of two proteins involved in functional Ca^2+^ transfer between the organelles, while SPLICS-based approach evaluates the juxtaposition of ER and mitochondria, giving mostly a structural information that can be suggestive of a functional readout in calcium signaling. The experiments display a complex behavior, suggesting the possibility that Sorcin might act specifically at the organelle interface to selectively fine-tune Ca^2+^-dependent signals.

These findings indicate that Sorcin is a novel, promising neurodegeneration marker, able to modulate calcium intracellular fluxes and may represent a trait of cellular stress dependent on neurodegeneration.

The effects due to Sorcin expression on calcium homeostasis, and in particular ER increased calcium levels, may provide a solution to the lack of adequate animal models for the study of brain aging and of neurodegeneration: the *Sri(-/-)* mouse model could represent one of the solutions looked after by the Alzheimer’s Association Calcium Hypothesis Workgroup^64^.

## Supplementary information

Supplementary figure legends

Supplementary Figure S1

Supplementary Figure S2

Supplementary Figure S3

Supplementary Figure S4

Supplementary Figure S5

Supplementary Figure S6

Supplementary Figure S7

Supplementary Figure S8

Supplementary Figure S9

Supplementary Figure S10

Supplementary Figure S11

Supplementary Figure S12

Supplementary Figure S13
