# Supplementary material for: Sorcin is an early marker of neurodegeneration, Ca2+ dysregulation and endoplasmic reticulum stress associated to neurodegenerative diseases
Source: Cell Death Dis. 2020 Oct 15;11(10):861. doi: 10.1038/s41419-020-03063-y (PMC7566454; doi:10.1038/s41419-020-03063-y)
Supplement: Supplementary file 1 — Supplementary figure legends [file 41419_2020_3063_MOESM1_ESM.docx]

**Supplementary figure legends**

**Figure S1**

(A): Immunofluorescence experiment showing that Sorcin is expressed in mouse cortex neurons (8 days in vitro) stained with rabbit anti-Sorcin and mouse anti-beta tubulin. Magnification: 63x, scale bar 20 µm (B): Sorcin partially colocalizes with RyR in cortical neurons (8 days in vitro) in ER; Sorcin is also localized in a more perinuclear region and, in addition, in the axonal region. Magnification 40x, scale bar 20 µm.

**Figure S2**

Sorcin (stained with mouse anti-Sorcin antibody 1:500) is present in microglia (A) and neurons (B, C), in the nuclear and perinuclear region of the cells, and in the axonal region. Magnification 40X (A), 63X (B,C), scale bar 20µm.

**Figure S3**

In order to have a control on the localization of Sorcin in vitro, we transfected customized egfp-sorcin plasmid (Invitrogen) in NSC-34 cells (Neuroblastoma X Spinal Cord hybrid cell line) (A, B) to overexpress the protein: in green, cells expressing EGFP-Sorcin showed nuclear, perinuclear and axonal-like staining of the protein; in blue nuclei stained with Hoechst. In red, endogenous sorcin staining with specific antibody against the protein; in blue nuclei: as shown in B, sorcin-Ab recognizes basal expression of the protein in all cells and red fluorescent signal (anti rabbit 594 Alexafluor Ab) was specifically increased in cells overexpressing the egfp-sorcin.

**Figure S4**

Immunofluorescence experiments showing the expression of RyRs in cortical neurons.

The Ryanodine receptor is expressed both in microglia (A) and in neuron (B, C) cells. Magnification 63X, scale bar 20µm.

**Figure S5**

RyR expression in cerebellar microglia cells. Cerebellar slices of mice expressing GFP (green) specifically in microglia, were stained with mouse anti-RyR antibody (red). Magnification 40x, scale bar 20µm.

**Figure S6**

Immunoprecipitation experiment: Sorcin and RyR coimmunoprecipitate in the presence of calcium from lysates of cortical neurons. The lysate was incubated with anti RyR (1:2000, Santa Cruz) pre-coupled with Protein G-Sepharose beads (30 μl). After 3 h incubation at 4 °C, beads were washed three times with ice cold buffer and centrifuged. Immunoprecipitated proteins were separated on 12% SDS-polyacrylamide gel and visualized by Western blot staining with anti-Sorcin antibody.

**Figure S7**

OneStep SPR experiment showing the interaction of Sigma1 receptor (1-138) immobilized on a COOH5 chip with human Sorcin in the presence of 1 mM EDTA.

In OneStep assays, Taylor dispersions were exploited to generate analyte concentration gradients that provide high-resolution dose response in single injections. Full analyte titrations were recorded over four orders of magnitude in concentration, up to 5 μM. The experiment are titrations of Sorcin at concentrations of 200 nM (green), 1.2 μM (blue) and 5 μM (black). The increase in RU relative to baseline indicates complex formation; the plateau region represents the steady-state phase of the interaction (RUeq), whereas the decrease in RU represents dissociation of analytes from immobilized ligands after injection of buffer. Kinetic evaluation of the sensorgrams (red lines) was obtained using the SensiQ Qdat program and full fitting with 1 site.

**Figure S8**

Western blot experiments representative of Sorcin expression in Figure 5A. Sorcin expression levels in SH-SY5Y cells upon 48h treatment with MPP+ and Rotenone, vs. control cells (non-treated, NT).

Cells treated with MPP+ (1 μM) and cells treated with Rotenone (100 nM) express increased amount of Sorcin with respect to control cells (p<0.05). Sorcin and β-actin were evaluated using rabbit homemade and Cell Signaling β-Actin mouse mAb #3700, respectively. The Sorcin/β-actin ratio, analyzed using Image Lab software that permits the normalization of a specific protein signal with the β-actin signal, is given for each lane.

**Figure S9**

Sorcin expression levels in substantia nigra of murine R6/2 model of HD vs. control mice. Western blot experiments representative of Sorcin expression in Figure 5B: 20μg of proteins were resolved on 12% SDS–PAGE and blotted; Sorcin and β-actin were evaluated using rabbit homemade and Cell Signaling β-Actin mouse mAb #3700, respectively. The Sorcin/β-actin ratio, analyzed using Image Lab software that permits the normalization of a specific protein signal with the β-actin signal, is given for each lane.

**Figure S10**

Western blot representative of Sorcin expression in Figure 6A.

**Figure S11**

Representative Western Blots of experiments in Figure 6B: band of Sorcin expression in samples from controls (CTRY, CTRO), patients with Down Syndrome (DS), patients with Down Syndrome with Alzheimer’s Disease (DSAD).

**Figure S12**

Western blot representative of Sorcin expression in Figure 7 (HeLa) and 8 (SHSY-5Y).

**Figure S13**

Representative images of SPLICS and PLA mito-ER experiments.
