# Supplementary figures and images for: Sorcin is an early marker of neurodegeneration, Ca2+ dysregulation and endoplasmic reticulum stress associated to neurodegenerative diseases
Source: Cell Death Dis. 2020 Oct 15;11(10):861. doi: 10.1038/s41419-020-03063-y (PMC7566454; doi:10.1038/s41419-020-03063-y)

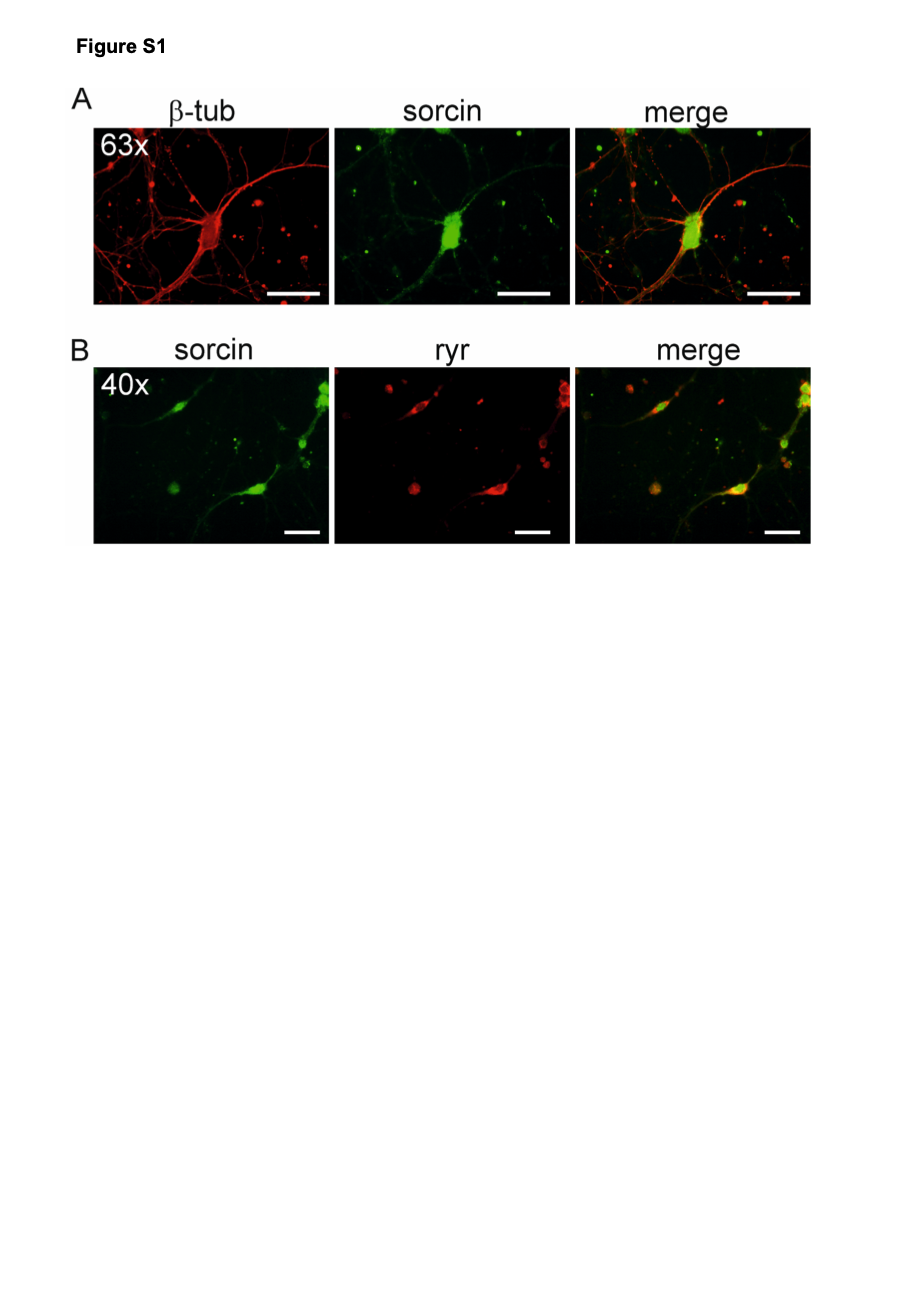

Supplement: Supplementary file 2 — Supplementary Figure S1 [file 41419_2020_3063_MOESM2_ESM.tif]

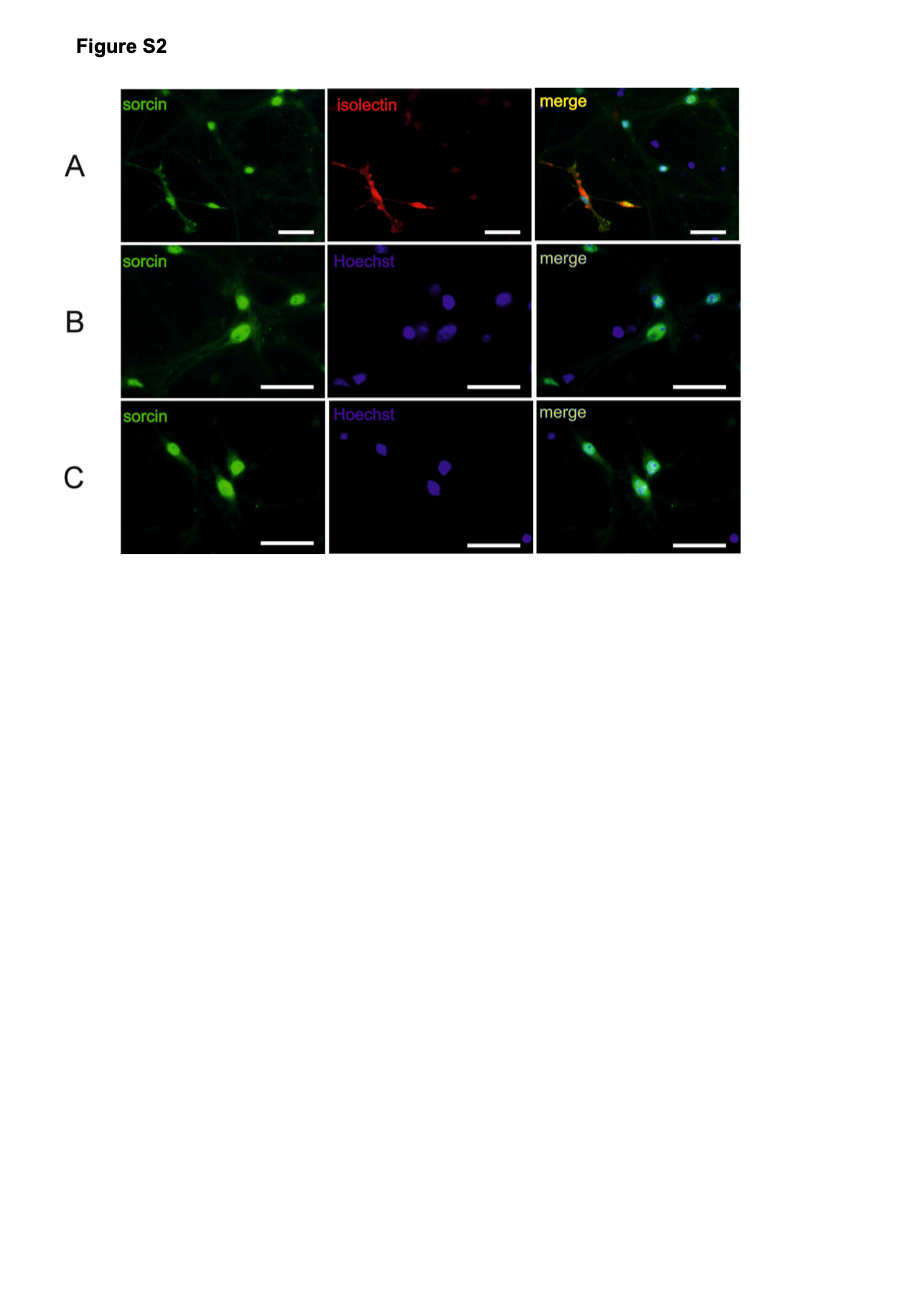

Supplement: Supplementary file 3 — Supplementary Figure S2 [file 41419_2020_3063_MOESM3_ESM.tif]

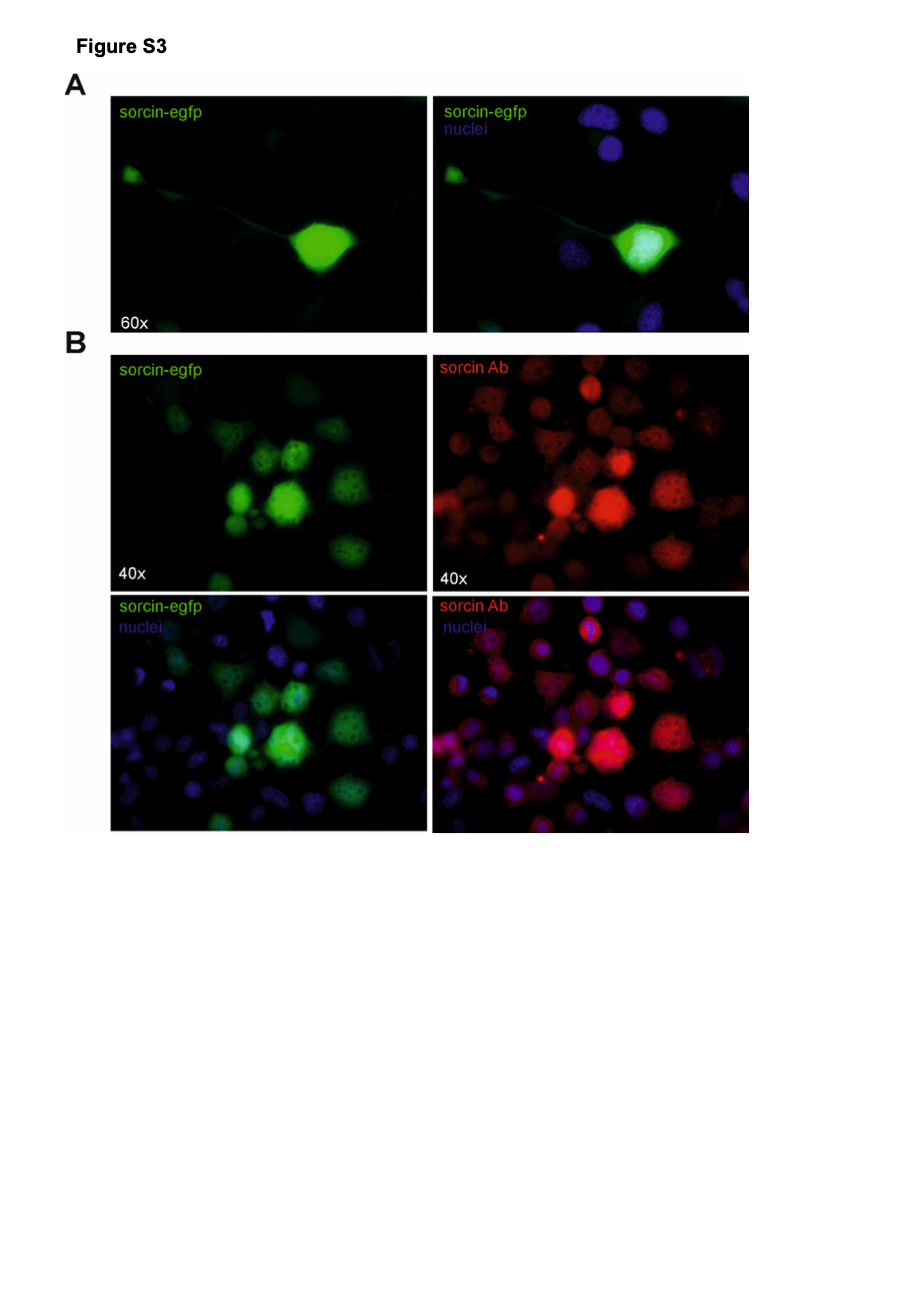

Supplement: Supplementary file 4 — Supplementary Figure S3 [file 41419_2020_3063_MOESM4_ESM.tif]

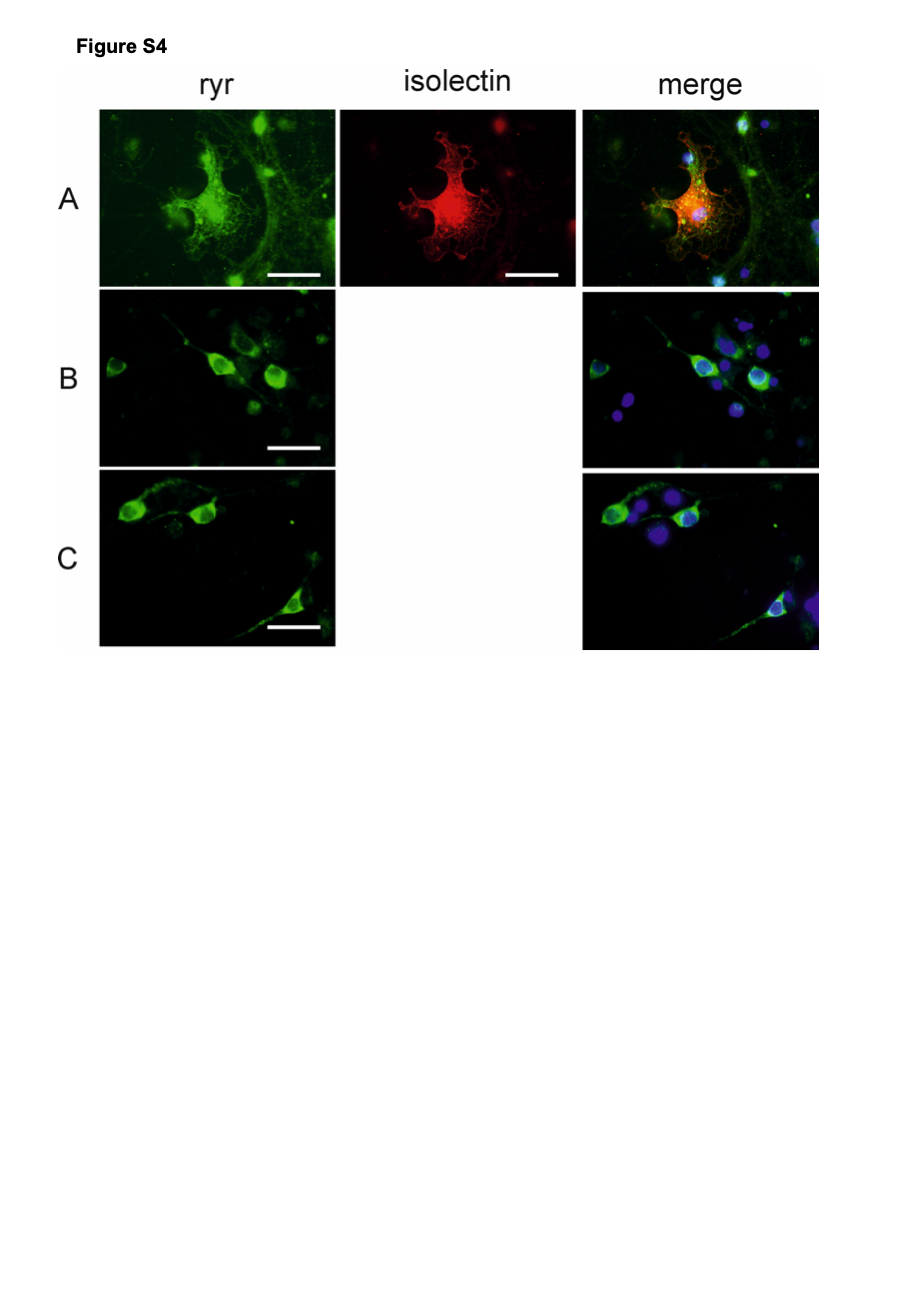

Supplement: Supplementary file 5 — Supplementary Figure S4 [file 41419_2020_3063_MOESM5_ESM.tif]

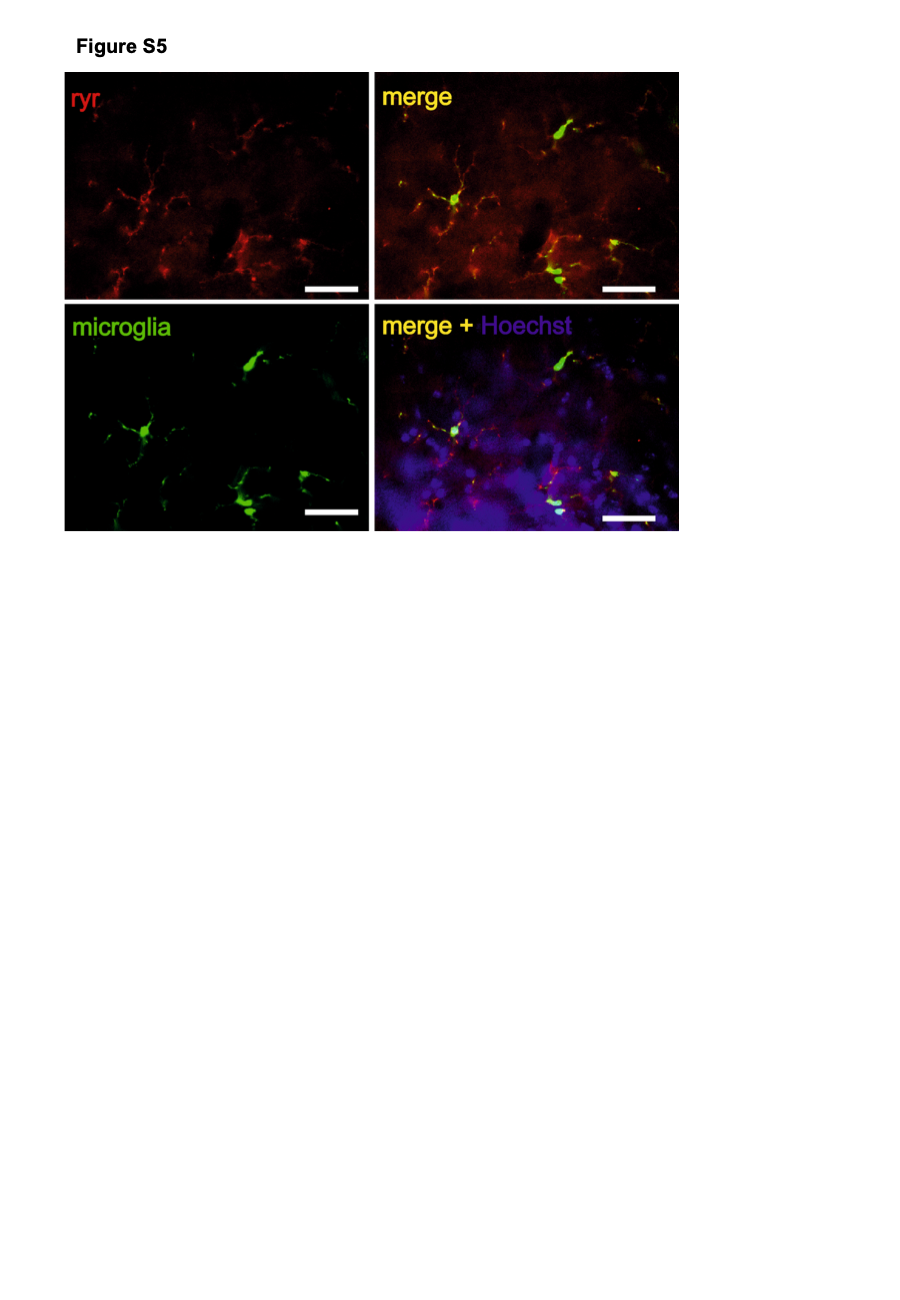

Supplement: Supplementary file 6 — Supplementary Figure S5 [file 41419_2020_3063_MOESM6_ESM.tif]

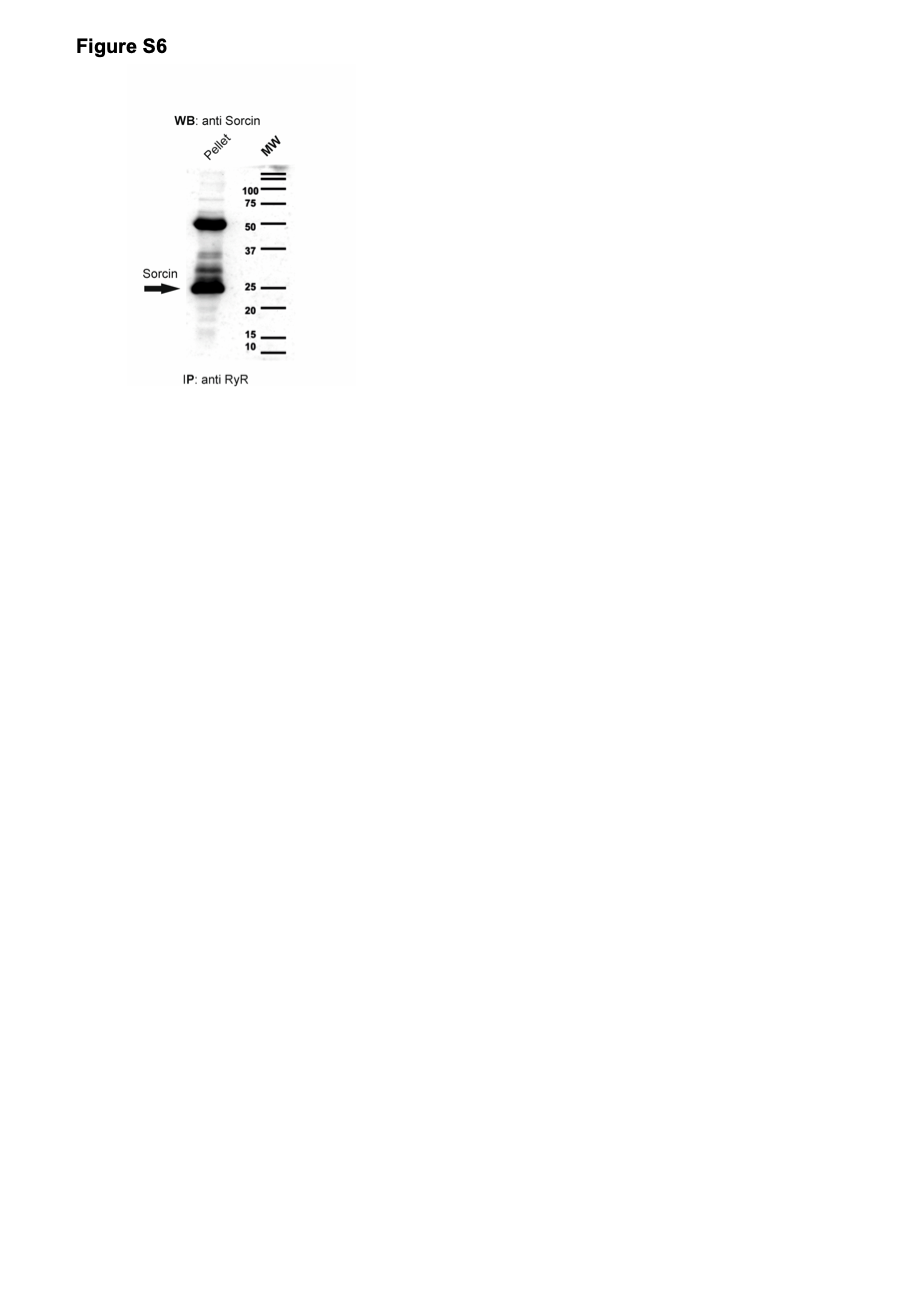

Supplement: Supplementary file 7 — Supplementary Figure S6 [file 41419_2020_3063_MOESM7_ESM.tif]

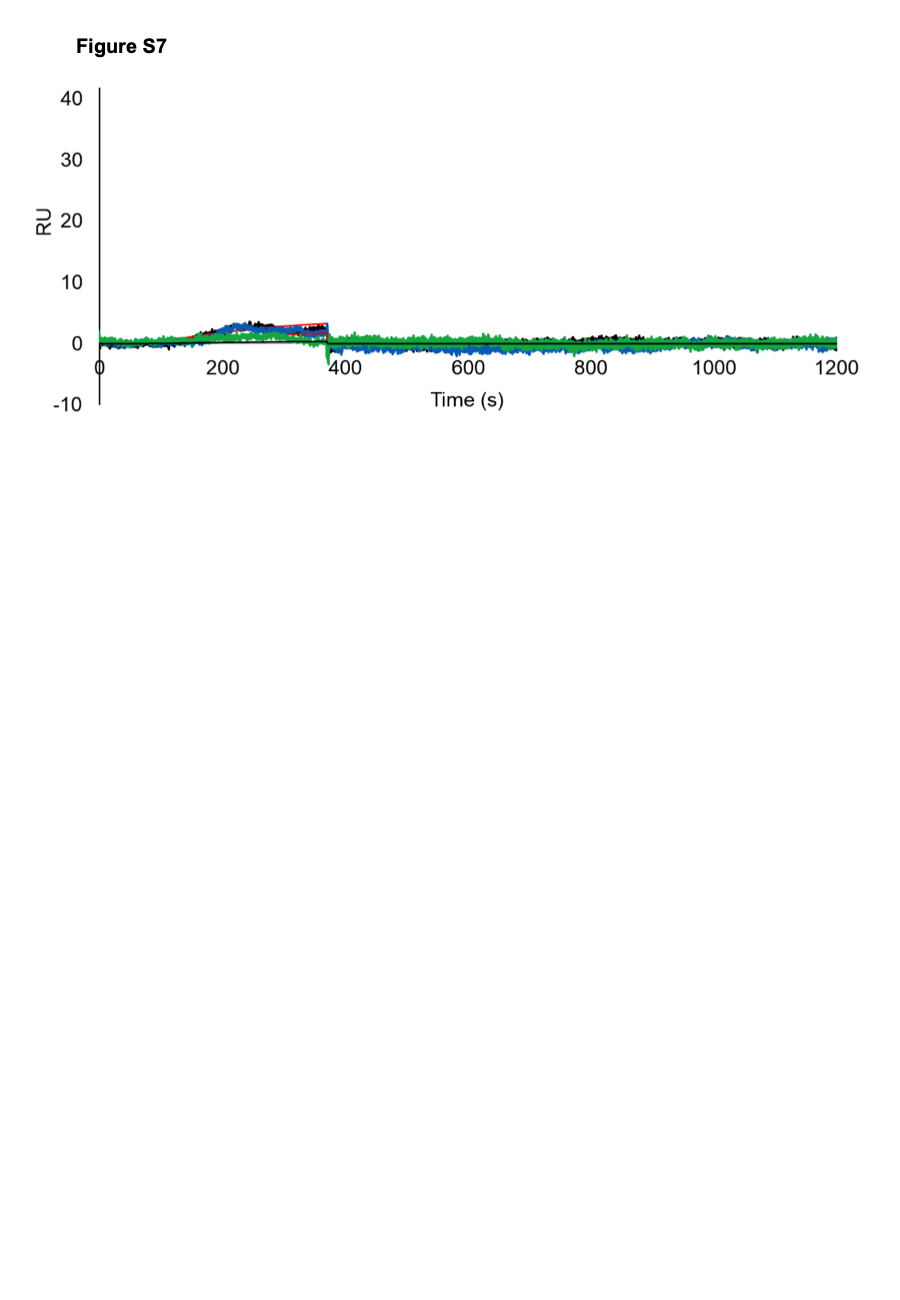

Supplement: Supplementary file 8 — Supplementary Figure S7 [file 41419_2020_3063_MOESM8_ESM.tif]

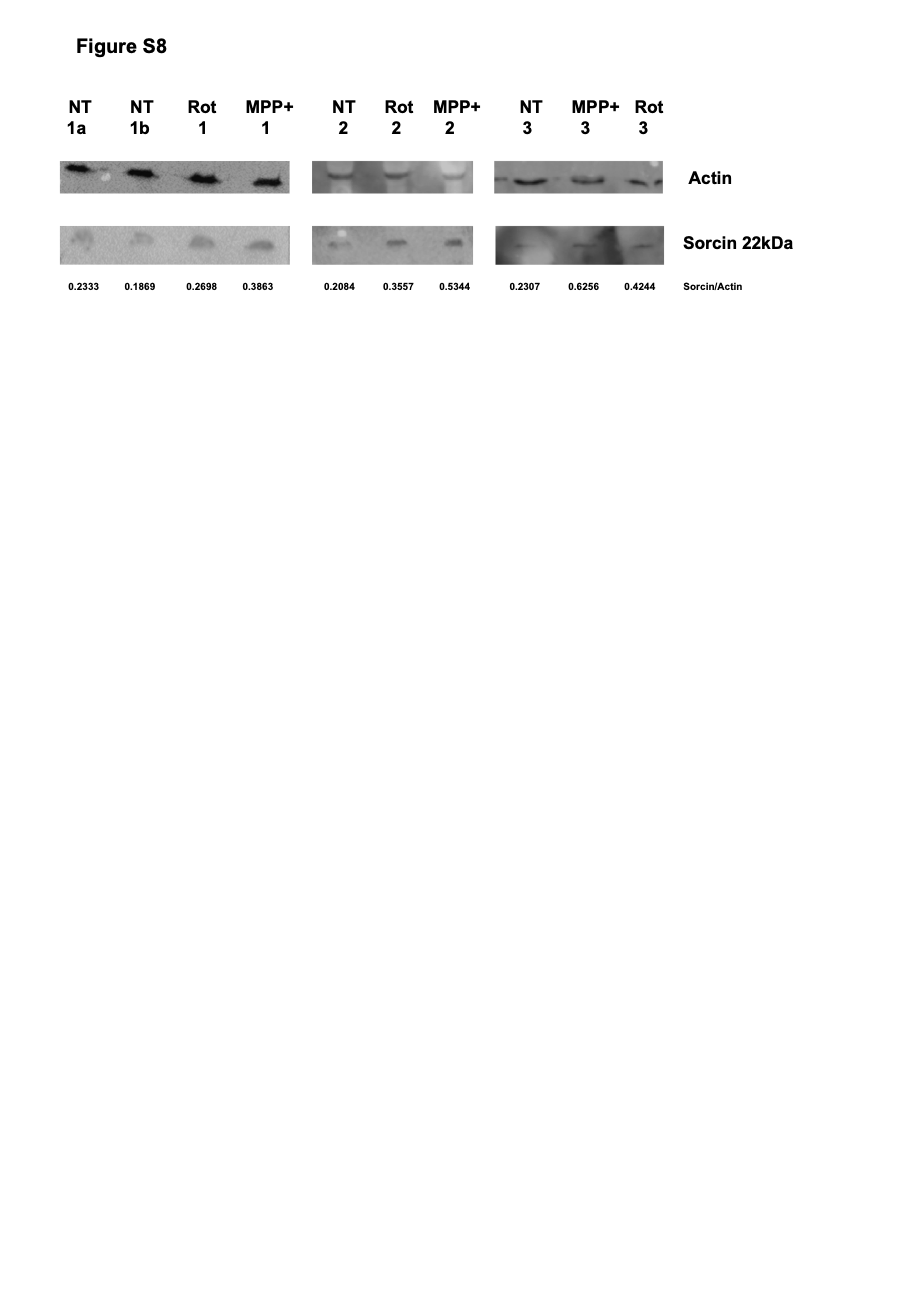

Supplement: Supplementary file 9 — Supplementary Figure S8 [file 41419_2020_3063_MOESM9_ESM.tif]

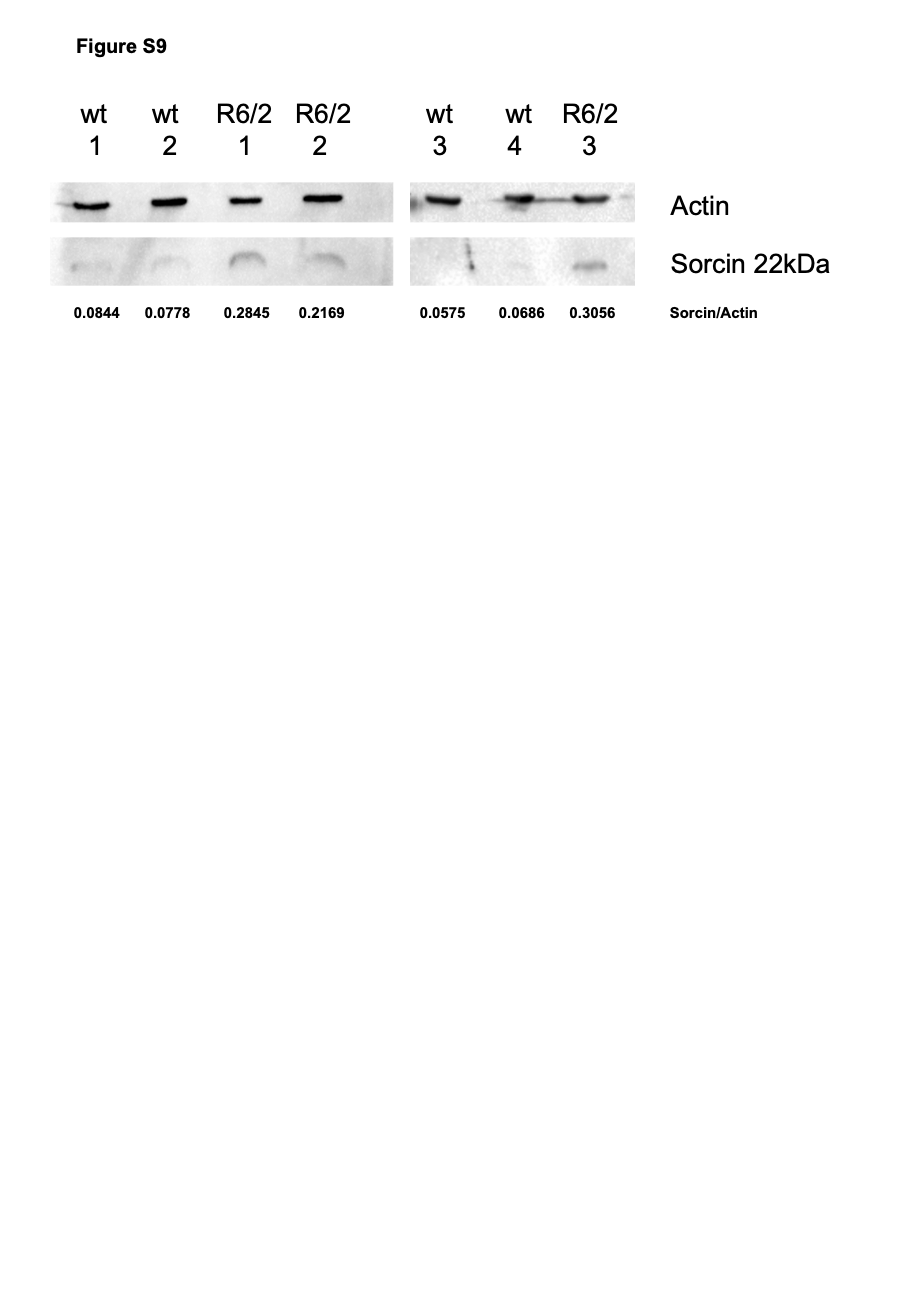

Supplement: Supplementary file 10 — Supplementary Figure S9 [file 41419_2020_3063_MOESM10_ESM.tif]

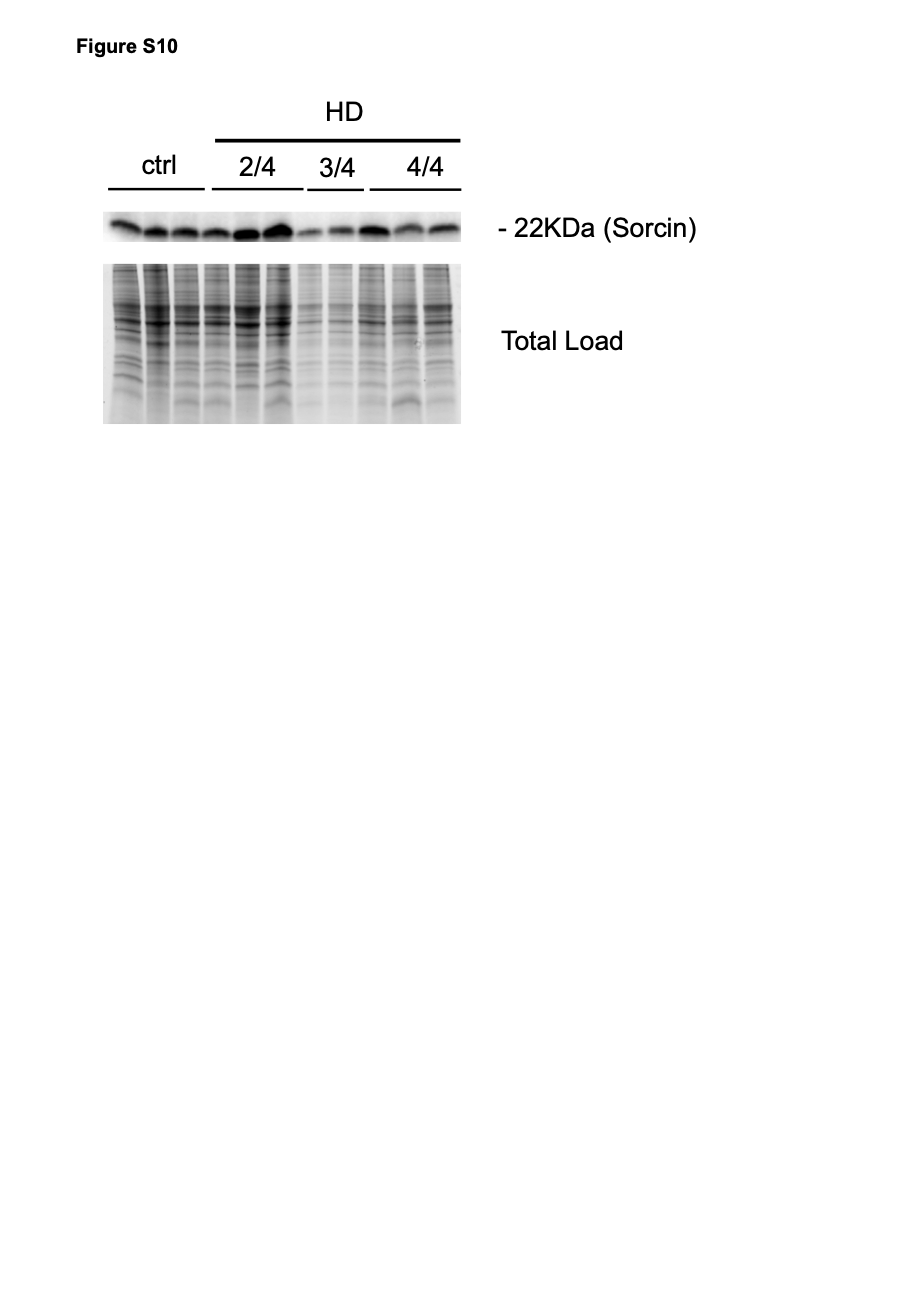

Supplement: Supplementary file 11 — Supplementary Figure S10 [file 41419_2020_3063_MOESM11_ESM.tif]

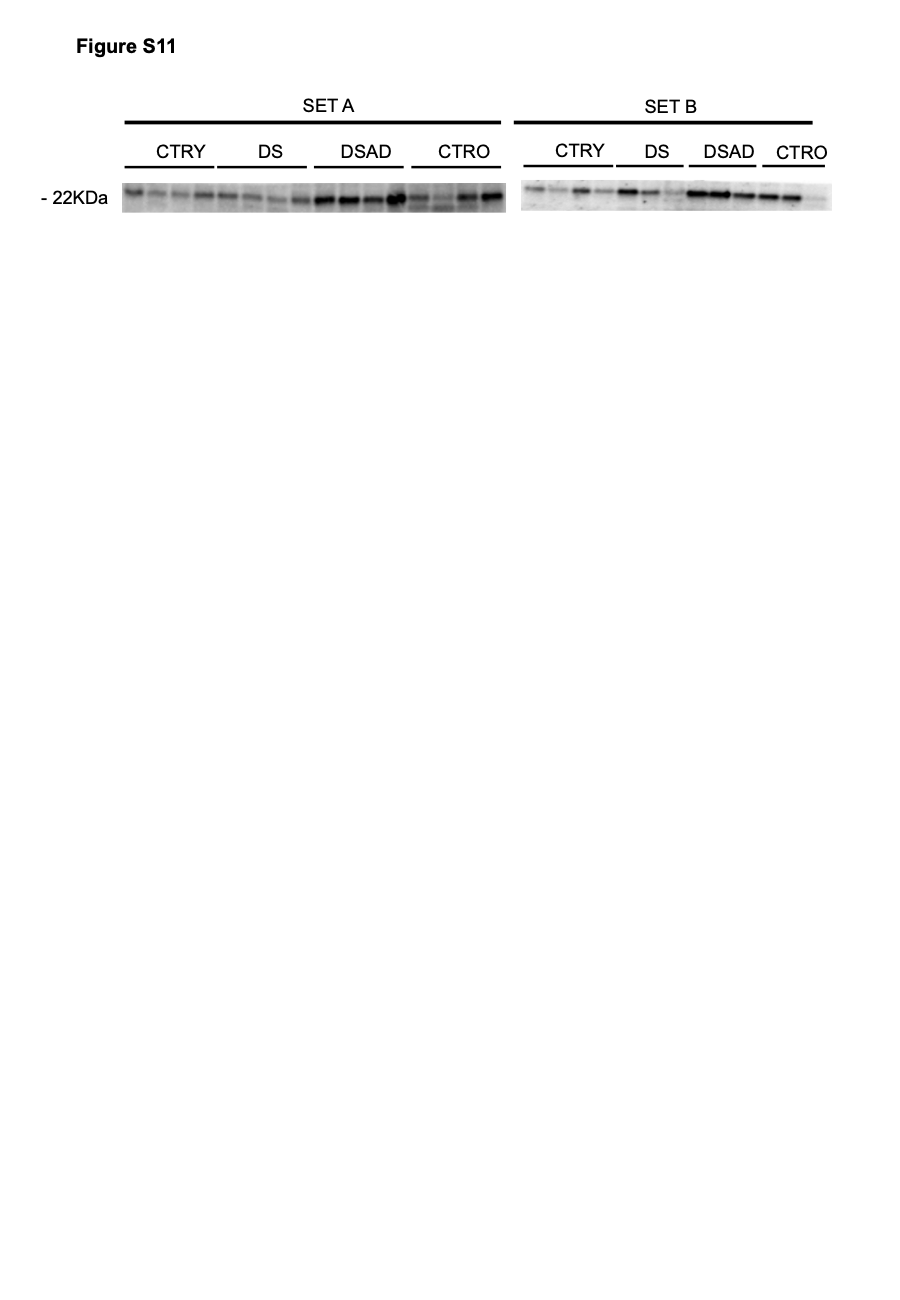

Supplement: Supplementary file 12 — Supplementary Figure S11 [file 41419_2020_3063_MOESM12_ESM.tif]

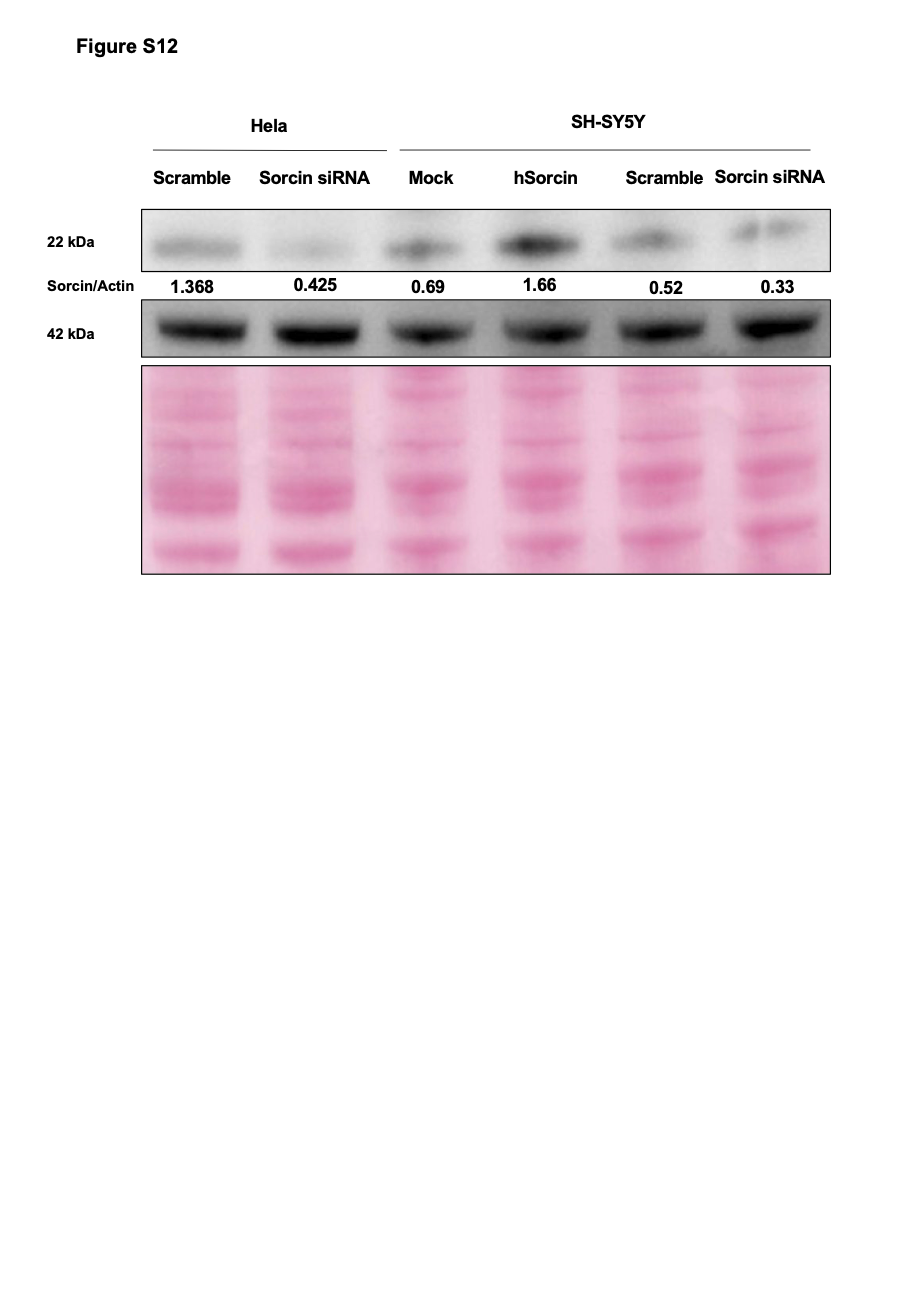

Supplement: Supplementary file 13 — Supplementary Figure S12 [file 41419_2020_3063_MOESM13_ESM.tif]

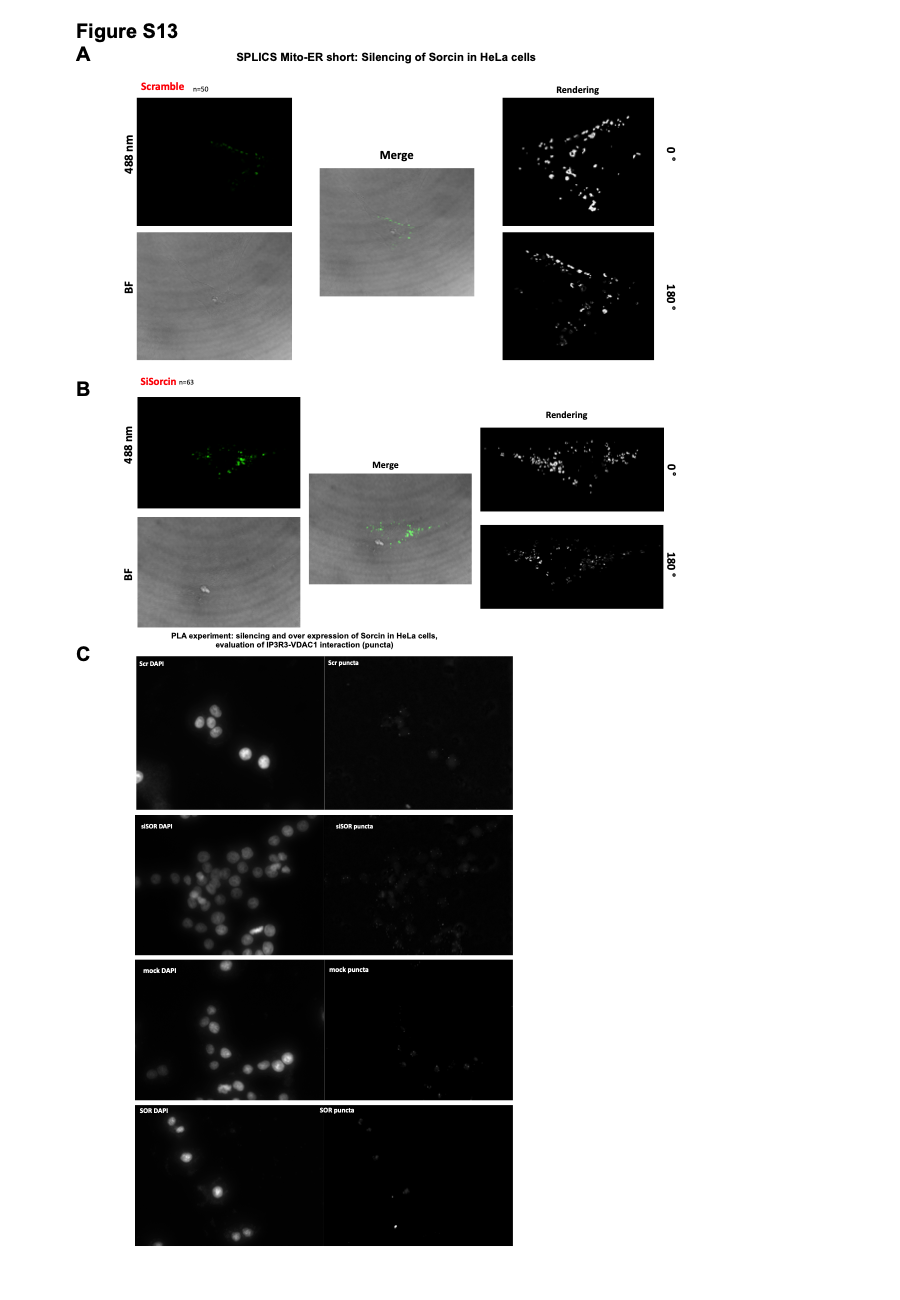

Supplement: Supplementary file 14 — Supplementary Figure S13 [file 41419_2020_3063_MOESM14_ESM.tif]
